# Effects of energy-matched low- versus high-carbohydrate diets on glycaemic control, lipid profile, and body composition in healthy adults: a systematic review and meta-analysis of randomised controlled trials

**DOI:** 10.1007/s00394-025-03862-z

**Published:** 2026-01-06

**Authors:** Alexandros Anagnostou, Eneko Larumbe-Zabala, Jo Fiore, Justin Roberts, Fernando Naclerio

**Affiliations:** 1 Institute for Lifecourse Development, School of Human Sciences, Centre for Exercise Activity and Rehabilitation, University of Greenwich, London, UK; 2 Department of Public Health, Fundación Canaria Instituto de Investigación Sanitaria de Canarias, Las Palmas, Spain; 3 Cambridge Centre for Sport and Exercise Sciences, Faculty of Science and Engineering, Anglia Ruskin University, Cambridge, UK

**Keywords:** Insulin resistance, Triglycerides, Energy-matched diets, Macronutrient distribution, Systematic review methodology

## Abstract

**Background:**

The comparative effects of energy-matched low-carbohydrate (LC) versus high-carbohydrate (HC) diets on metabolic and anthropometric outcomes in healthy adults remain unclear.

**Objective:**

To evaluate the effects of LC diets (≤ 44% of total daily caloric intake [TDCI] from carbohydrate) versus HC diets (≥ 45% TDCI) on fasting glycaemia, insulinaemia, blood lipids, and body composition in non-medicated, disease-free adults under energy-matched conditions.

**Methods:**

Randomised controlled trials (RCTs) were identified through systematic searches of PubMed and secondary sources up to April 2025. Eligible studies compared energy-matched dietary interventions and reported pre- and post-intervention data for fasting blood glucose (FBG), fasting insulin (FINS), blood lipids (total cholesterol [TC], HDL-cholesterol [HDL-C], LDL-cholesterol [LDL-C], triglycerides [TAG]), and/or body composition. Pooled effect sizes (Hedges’ g) and 95% confidence intervals (CIs) were calculated using a random-effects model.

**Results:**

Eighteen RCTs involving 905 participants met the inclusion criteria. LC diets produced greater reductions in FBG (*g* = − 0.364; 95% CI − 0.709 to − 0.019; *P* < 0.001) and FINS (*g* = − 0.190; 95% CI − 0.361 to − 0.014; *P* = 0.034) compared with HC. TAG decreased (*g* = − 0.379; 95% CI − 0.540 to − 0.219; *P* < 0.001), and HDL-C increased (*g* = 0.389; 95% CI 0.229 to 0.550; *P* < 0.001) under LC diets. HC diets led to a greater reduction in LDL-C (*g* = −  0.225; 95% CI − 0.406 to − 0.043; *P* = 0.009). No significant effects were found for TC. LC diets also reduced body mass (*g* = − 0.183; 95% CI − 0.349 to − 0.017; *P* = 0.031) and fat mass (*g* = − 0.304; 95% CI −  0.548 to − 0.059; *P* = 0.015) to a greater extent than HC, with no effect on fat-free mass.

**Conclusion:**

Under energy-matched conditions, LC confers modest advantages for glycaemia, HDL-C, and TAG, whereas HC better lowers LDL-C. Most effects do not depend on exercise status, offering evidence to guide carbohydrate intake recommendations in diets where total caloric intake remains unchanged.

**Supplementary Information:**

The online version contains supplementary material available at 10.1007/s00394-025-03862-z.

## Introduction

Obesity represents a significant global health challenge, with projections indicating that by 2035, over 50% of the world’s population could be classified as overweight or obese [1]. The resulting economic burden due to obesity-related healthcare and societal costs is substantial [1, 2]. Obesity significantly increases the risk of developing cardiovascular disease, type 2 diabetes, and various cancers, highlighting the need to understand the underlying metabolic mechanisms, particularly insulin resistance and hyperinsulinemia [3–6]. Hyperinsulinemia represents a critical target for investigating dietary strategies aimed at reducing the risk of metabolic syndrome, given its assssociation with carbohydrate-induced insulin secretion and its strong link to obesity and metabolic syndrome development [7].

Moreover, recent reviews have questioned the long-standing association between saturated fat intake, elevated cholesterol levels, and increased cardiovascular disease risk. These analyses found insufficient evidence to support this association. Conversely, some findings suggest that higher intake of saturated fats may be beneficial to health, particularly when dietary fats substitute carbohydrates. Such substitution of increased fats and reduced carbohydrates has been shown to improve lipid profiles by reducing the total cholesterol: HDL-C and LDL-C: HDL-C ratios [8–11].

Metabolic syndrome is diagnosed when an individual presents with at least three of the following clinical markers: central obesity (waist circumference ≥ 102 cm in men or ≥ 88 cm in women), elevated triglyceride (TAG) levels (≥ 1.7 mmol/L), reduced HDL cholesterol (< 1.0 mmol/L in men or < 1.3 mmol/L in women), elevated blood pressure (≥ 130/85 mmHg), or elevated fasting plasma glucose (≥ 5.6 mmol/L), or is receiving treatment for any of these conditions [12].

The available evidence suggests that the macronutrient distribution range, not merely total caloric intake, plays a critical role in regulating blood lipid profiles, insulin and glucose levels, body composition, and the development, remission, and prevention of metabolic disorders. Extensive research has examined the effects of various macronutrient distribution ranges [13, 14], particularly carbohydrate intake, on metabolic markers [15–17] and body composition [18, 19]. However, significant methodological heterogeneity among these studies has hindered the ability to reach a consensus and formulate evidence-based dietary recommendations for the general healthy adult population. For instance, regular physical activity is effective in managing dyslipidaemia and may serve as a primary prevention strategy, potentially reducing the need for pharmacological intervention [20]. Furthermore, existing literature supports the role of exercise as an effective intervention for glycaemic control and enhancement of insulin sensitivity, not only in healthy, non-overweight individuals but also in obese, prediabetic, and diabetic populations [21–25].

Therefore, this systematic review and meta-analysis aimed to quantify the effects of dietary carbohydrate modification on metabolic health and body composition in healthy, non-medicated adults with or without regular physical activity. We specifically compared recommended carbohydrate intakes (≥ 45% of total daily caloric intake, TDCI) [26–28] against lower carbohydrate intakes (≤ 44% TDCI) in energy-matched conditions. Primary outcomes included changes in fasting blood glucose, fasting insulin, and blood lipid profiles (TC, HDL-C, LDL-C, and TAG). Secondary outcomes assessed changes in body composition parameters: body mass (BM), fat mass (FM), and fat-free mass (FFM). The findings aim to provide robust evidence to inform dietary recommendations and potentially contribute to public health strategies for reducing the global burden of obesity and metabolic syndrome.

## Methods

This study was performed under the Preferred Reporting Items for Systematic Review and Meta-Analysis (PRISMA). The analysis method and inclusion criteria were specified and documented in a protocol registered at the International Prospective Register of Systematic Reviews, PROSPERO (CRD420251012019).

### Search strategy

A systematic review of the literature was conducted in accordance with PRISMA guidelines [29–31] and the guidelines described for systematic reviews in the nutrition field [32]. The keywords used to ensure that all potentially eligible studies were identified in our search included: (energy-matched or isoenergetic or conventional or western or traditional or vegan or high* protein or low protein or vege* or vega* or high* carb* or low carb* or high* fat or low fat or Mediterranean or keto* or carnivore or diet) and (body composition or lean body mass or LBM or fat-free mass or FFM or blood markers of health or fasting glucose or triglycerides or total cholesterol or high density cholesterol or HDL or low density cholesterol or LDL or total leukocytes or leukocyte subsets or neutrophils or eosinophils or basophils or monocytes or lymphocytes or platelets or physical performance or upper body strength or lower body strength or 1 repetition maximum test or 1-RM or markers of physical performance or 10-m walking or 10-MW or 6-min walking or 6-MW or 5-times sit-to-stand or 5-STS or 30-s sit-to-stand or 30-STS or timed up-and-go or TUG tests) or (documented dietary habits or food tracking or macronutrient tracking or food diary or MyFitnessPal or diet tracking) not (chronic or injury or disease or metabolic syndrome or diabet* or drugs or medications or adolescence or acute or Illn* OR Canc* OR Frail* OR Sick* OR ICU OR Sclerosis OR Patient* OR Hospit* OR Rehab* OR Child* OR Kids* OR Toddler* OR Animal* OR Rats* OR Mice OR Mouse or short-term or days or acute or osteoporosis or hiv or rehabilitation or morbid obesity or sarcopenia).

**Fig. 1:**
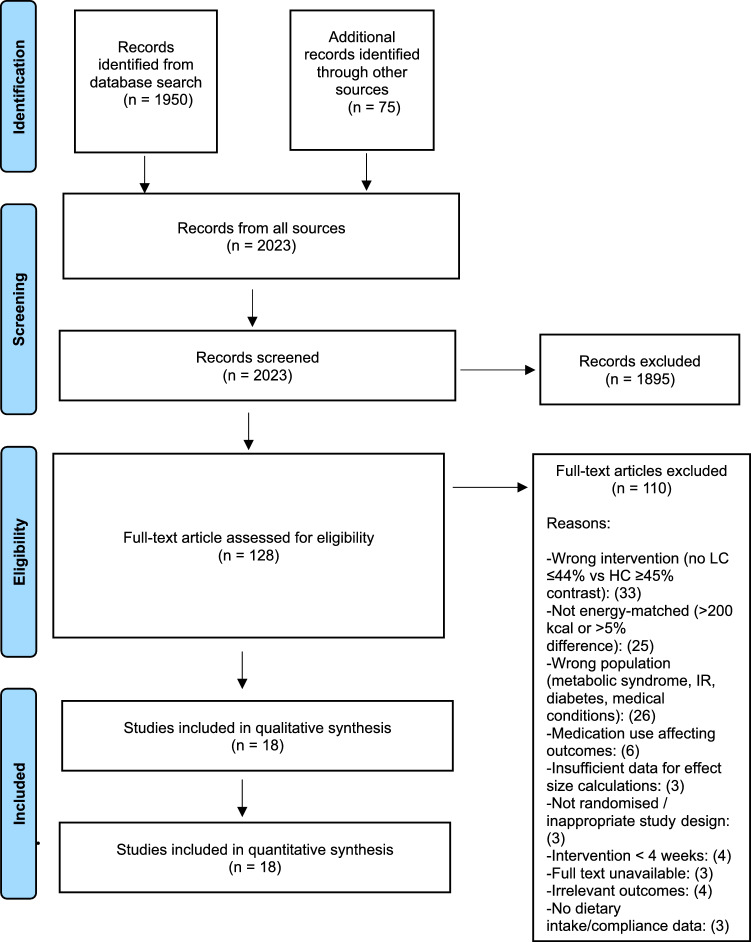
PRISMA-P Flow chart diagram of the study selection

The search was conducted between July to December 2024. The initial result from the PubMed database was 62,032 studies before filters were applied. Following the automatic filter application of PubMed to make the search accurate, the result was reduced to 1950. After hand-picking the eligible studies based on title and published abstract, we selected 53 studies from the search in PubMed and another 75 found through references of similar SR and MA. Finally, the researcher (AA) selected 18 studies eligible for data extraction, and the remaining were excluded due to failing our eligibility criteria, incomplete data, or variables measured that were not relevant to our review (Fig. 1). A second reviewer, FN, independently screened the identified publications before proceeding to the data extraction.

### Eligibility criteria

The inclusion and exclusion criteria were the following: (i) Randomised Controlled trials only; (ii) with healthy human adults, including overweight and obese, not injured, not rehabilitating, not under any medication, disease-free and (iii) to ensure the inclusion of metabolically healthy, non-diabetic populations, studies that explicitly recruited insulin-resistant or metabolically impaired participants were excluded [33–35]. We assessed whether they had IR based on one of the following scenarios: studies had specifically tested for IR, using methods such as HOMA-IR [36, 37] or explicitly mentioned in their eligibility criteria that they included only non-diabetic participants, or in some cases classified their participants as having no existing medical conditions. Minor variations in insulin sensitivity within the normal physiological range were accepted, as such fluctuations are common among free-living healthy adults and do not indicate the presence of diabetes or metabolic impairment. When we observed contradicting indications from their baseline blood tests, we excluded the study. In addition, (iii) participants had to be: not pregnant and/or not diagnosed with metabolic syndrome (studies with three or more metabolic syndrome [MS] risk factors present at baseline measurements were excluded, even if the study classified the participants as healthy and/or not diagnosed with MS) [38]; (iv) a minimum of two intervention groups was required in each publication, comparing a traditional diet $\ge$ 45% CHO (HC) of a TDCI to a low CHO ≤ 44% (LC), based on the minimum recommended intake of $\ge$ 45% set by the Institute of Medicine and supported by the American Health Associations along with the governments of the U.S. and U.K. [26, 27, 39, 40]; (v) the dietary interventions had to be energy-matched and any study with > 200 kcal or > 5% difference between groups was excluded [41, 42]. In this context, ‘energy-matched’ refers to interventions in which both comparison groups were designed to maintain equivalent total energy intake across the study period, although not necessarily identical to participants’ habitual or baseline caloric intake. This approach ensured that the observed effects reflected differences in macronutrient composition rather than disparities in overall energy consumption. We used the former criterion for studies with groups consuming a mean daily caloric intake of ≥ 1500 kcal and the latter for studies with diets of < 1500 kcal. This decision was made based on the evidence that variations in daily caloric intake, especially if it leads to a caloric deficit, result in weight loss, affect blood lipid profile, along with fasting insulin [43, 44]. Furthermore, we decided to use a variation of 200 kcal from the target TDCI between diet groups as the cutoff point, similarly to other publication [45] which defines the groups as energy-matched as aligned with previously published research [46, 47]; (vi) in addition, dietary intake should have been reported and monitored along with dietary advice provided to the participants to ensure compliance; (vii) the minimum duration of intervention was 4 weeks; and (viii) lastly, measured variables should be sufficiently presented as mean differences and standard deviation (SD) or to allow for calculations of mean and SD for some or all of the following dependent variables: TC (mmol/l), HDL-C and triglycerides (mmol/l), insulin (mmol/l), testosterone (ng/dl), cortisol (μg/dl), BM (kg), FM (kg), body fat (%), FFM (kg) and muscular strength (1RM).

In accordance with the PICOTS framework, the review was structured as follows:

- Population: Healthy, non-diabetic, metabolically healthy adults, including those who were overweight or obese.
- Intervention: Low-carbohydrate diets providing ≤ 44% of total daily caloric intake (TDCI) from carbohydrates.
- Comparator: High-carbohydrate diets providing ≥ 45% of TDCI.
- Outcomes: Cardiometabolic, hormonal, and body-composition indicators, including total cholesterol (TC), HDL-cholesterol (HDL-C), triglycerides (TG), insulin, testosterone, cortisol, body mass, fat mass, body-fat percentage, fat-free mass (FFM), and muscular strength.
- Timing: Interventions lasting a minimum of four weeks.
- Setting: Controlled laboratory or free-living conditions, as specified by the original study authors

### Study records

#### Data management and selection

(i) The initial identification of data from the selected studies was conducted by screening the titles and published abstracts; (ii) full-text articles were then retrieved and assessed for eligibility; (iii) in certain cases, authors were contacted to obtain precise data that were only available in graphical form or not reported as means (± SD), or in any other usable format for calculation; (iv) when necessary, SDs were calculated from mean confidence intervals (MCI), standard errors (SE), or percentage changes in mean and SD.

#### Data collection process and coding

The extracted qualitative and quantitative data from each study were as follows: (1) researcher’s name; (2) year of publication; (3) population characteristics at baseline; (4) dietary intervention and exercise intervention when applicable; (5) study duration; (6) methods of monitoring compliance; (7) sample number per group; (8) pre and post measurements expressed in means and SD for the following variables: (i) total cholesterol, lipoproteins and triglycerides; (ii) blood glucose; insulin (iii) waist circumference, body fat (BF), body fat % (BF%), lean mass (LM)/ FFM, total mass/BM; (iv) blood pressure; and (v) V̇O_2max_; lower and upper body strength (expressed in kilograms).

### Risk of bias in individual studies

Methodological details concerning the potential influence of bias were carefully evaluated. As part of the quality control process, two independent reviewers (AA and FN) individually extracted information from each study. The risk of bias for each study was assessed using six domains from the Cochrane collaboration tool [48], which included: randomisation process, period of carryover effects, deviations from intended intervention, missing out data, measurement of the outcome, selection of the reported results, and overall bias. These domains corresponded to various types of bias, including selection, allocation, performance, detection, attrition, reporting, and others. The two reviewers conducted independent evaluations of study quality, comparing and discussing their results until reaching a consensus [If a consensus could not be agreed, a third reviewer (JF) was consulted]. Each domain was rated as − 1 for high risk, 0 for unclear risk, and 1 for low risk, with total scores ranging from − 6 to 6.

### Data analysis

A meta-analysis was conducted using Comprehensive Meta-Analysis (CMA) V4 software under the condition that the included studies exhibited no significant heterogeneity (*p* > 0.10 and I^2^ < 50). A random-effects model was selected to account for potential variability in the true effect sizes across studies. To perform the analysis, at least four studies per outcome were necessary to calculate weighted group mean differences, 95% confidence intervals (CIs), and heterogeneity p-values. Data on pre- and post-intervention means, standard deviations (SD), and sample sizes were extracted for both the HC and LC groups. When SDs were not directly provided, but standard error, CI 95%, mean change, or percentage change was available, SDs were calculated accordingly. Hedges' g was used to determine effect sizes, interpreted as small (0.2), medium (0.5), and large (0.8) effects [49]. The primary analysis focused on comparing the effects of HC versus LC diets on various outcomes, including total cholesterol, HDL-C, LDL-C, TAG, BM, FFM, FM, upper and lower body strength (1RM). When quantitative analysis was not feasible, findings were summarised descriptively. If heterogeneity or sensitivity analyses indicated high variability, further meta-analysis was avoided, and results were presented individually. Outliers were identified by analysing standardised residuals beyond the ± 1.96 range. Publication bias was assessed through funnel plots (effect size vs. standard error), the “Trim and Fill” method for random effects, and Orwin’s Fail-Safe N analysis.

## Results

### Study selection

The search strategy is outlined in Fig. 1. An initial search yielded 1,950 potentially relevant references. After reviewing the titles and abstracts, 53 publications were selected for further evaluation. Following this evaluation, 48 studies were excluded, leaving a total of 5 studies [37, 43, 50–52]. Additionally, through references from similar systematic reviews and RCTs, 75 more studies were identified, of which 13 were selected [36, 47, 53–63] and fully reviewed, providing a total of 18 studies for the final meta-analysis. Table S15, summarising the 110 full-text articles assessed and subsequently excluded with reasons for exclusion, is provided in the Supplementary Material.

### Characteristics of the included studies

The included studies were generally of high methodological quality, with a low risk of bias as assessed using the Cochrane tools (ROB2_IRPG_beta_v9 for parallel-group designs and ROB2_crossover_beta_v2 for crossover trials). Table S14 (Supplementary Material) summarises the individual risk of bias assessments. Eighteen studies reported valid HC or LC group data, comprising 905 participants (male and female, aged 18–70 years).

Sample sizes ranged from 7 to 138 participants, with similar demographic characteristics within studies. The HC diet groups included 5 to 60 participants, while 6 to 59 subjects were assigned to the LC group. The selected RCTs followed a parallel design [37, 43, 50, 52–55, 57–63], a randomised crossover design [47, 51, 64], or a controlled 3-way crossover design [56].

All eighteen included studies compared energy-matched dietary interventions, with inter-group energy intake differences of ≤ 200 kcal or ≤ 5% of TDCI, contrasting diets providing ≥ 45% vs ≤ 44% of TDCI from carbohydrates.

The methods utilized by the researchers to ensure compliance and accuracy included: TDEE calculation equations, food diaries, diet analysis by software, provided measuring tools, utensils, and digital scales for portion size, handouts with list of foods, meal plans and recipes, specific to each diet group were shared, educational meetings, counselling, and supervision with dieticians, supplementation, urine tests (24 h urine nitrogen/urea/ketone levels), blood ketone levels, blood glucose, regular weigh-ins, all the meals prepared, leftovers returned and measured for accuracy of TDCI.

Several of the included studies employed various methods to calculate the total daily energy expenditure (TDEE), resting metabolic rate (RMR), or basal metabolic rate (BMR) to appropriately tailor the dietary interventions [37, 51, 52], predictive equations [43, 50] and stable isotope analysis [56]. Body composition was measured via various methods, including Dual X-ray absorptiometry (DXA) [37, 43, 50, 51, 53, 54, 57, 62], Bioelectrical Impendence [52, 61], and Digital Scales [60].

Table 1 provides a structured overview of study design, dietary macronutrient distributions, primary outcomes, and key findings in a format that enhances readability while preserving methodological detail for reproducibility; the accompanying GRADE ‘Summary of Findings’ table (Supplementary Table S15) summarises the certainty of evidence across all primary metabolic and anthropometric outcomes.

**Table 1:** Summary of the included Studies

Study	Design	Sample	Duration	Energy Distribution	Diet composition (mean % CHO/PRO/FAT)	Exercise	Outcomes
Wilson et al. [50]	PG	N = 25; Men; Age = 18–30; Normal weight	11 wks	Maintenance calories ≈ 2600 kcal determined by the Mifflin St. Jeor equation	HC (55/20/25); LC (5/20/75) CHO reintroduced for the KD at weeks 10 and 11	RT, 8 wks, 3 d/wk, 5 to 11 exercises per session; Load increasing weekly 65–95% 1RM and reps deceasing from 15 to 5; Week #1–4 hypertrophy, Week #5–8 maximal strength	↑LBM wk 0-10^a^; ↑LBM wk 10-11^f, h^; ↑Wingate PP^b^; ↑SQ & BP^a^; ↔ TC, HDL, LDL; ↑TAG^c^ wk 10–11; ↑Total Testosterone^c^; ↔ Fasted insulin
Parr et al. [43]	PG	N = 89; Men and Women; Age = 35–59; Overweight/Obese	16 wks	Reduced 250 kcal/d from maintenance ≈ 1600 kcal; citing that they estimated it based on the SR by Frankenfield et al. [65]	HC (55/30/15); LC (40/30/30)	RT × 3/wk (48 sessions in 16 wks; Individualised programs based on 1RM and Endurance exercise × 4/wk = 250 kcal/day	↓BF^a^; ↑LBM^a^; ↑V̇O_2max_ and 1RM^a^; ↓HOMA-index^a^
Cornier et al. [37]	PG	N = 21; Women; Age = 23–53; Obese	16 wks	Reduced 400 kcal/d from maintenance; Estimates of daily energy intake were made using 3-day food diary, 3-day control diet, and baseline RMR plus an activity factor	HC (60/20/20); LC (40/20/40)	TDEE was not measured; PA from interviews and questionnaires (unspecified) was tracked to make sure it was the same throughout	↓BM^a, e, g^; ↓Fasted insulin^a^; ↔ TC, HDL, LDL, TAG
Paoli et al. [52]	PG	N = 19; Men; Age = 20–40; Normal weight	8 wks	Maintenance calories ≈ 3500 kcal; The caloric intake of the dietary patterns provided was calculated by assigning an energy expenditure of 45 kcal/kg of muscle mass [49]	HC (55/25/20) LC (5/25/70)	Competitive Body building preparation	↓FM^C^; ↑LBM^b^; ↑1RM^a^; ↑REE^b^; ↓TC^c^; ↑HDL^c, e^; ↓TAG^c, f^; ↓Glucose and Insulin^c, e^; ↓Testosterone in KD^c^: ↓IGF-1^c^; ↓IL-6^c^, ↑IL-6^b^; ↓TNF-Α^c, e^; ↑Max strength (1RM)^a^
Layman et al. [54]	PG	N = 24; Women; Age = 45–56; Overweight/Obese	10 wks	Reduced ≈ 400 kcal/d from maintenance; ≈ 1663 kcal/day	HC (58/16/26); LC (41/30/29)	Physical activity was monitored with questionnaires and was kept constant for each subject throughout the study	↑ Fat: Lean mass loss^a, d, h^;↓TC^a^; ↓ LDL^a^; ↔ HDL; ↓ TAG^c^; ↔ FINS; ↔ FBG
Brehm et al. [57]	PG	N = 42; Women; Age = 29–59; Overweight/Obese	24 wks	Baseline: HC (1707 kcal); LC (170 kcal)Mo 3: Hypocaloric; HC (1420 kcal); LC (1119 kcal)Mo 6: Hypocaloric; HC (1373 kcal); LC (1273 kcal)	HC—3mo (54/18/28), 6mo (53/18/29); LC—3mo (15/28/57), 6mo (30/23/46);	Participants continued with their baseline level of activity. Unspecified	↓BM^a, f, h^; ↓FM^a, e, h^; ↓FFM^a, e, h^; ↓TAG^a, d, h^; ↓LC^a^; ↓LDL^a^; ↑HDL^a^; ↓FBG^a^; ↓FINS^a^ (Significantly more for LC at 3 vs 6 mo)
Walberg et al. [59]	PG	N = 12; Women; Age 23–36; Obese	5 wks	28-d liquid diet ≈ 530 kcal/d; 330 kcal from formula: 33 g protein, 44 g C, 3 g fats; The remaining 200 kcal was added as C or Fats accordingly	HC (71/25/4); LC (33/25/42)	Brisk walk or jogging 30-45 min at 60% V̇O_2max_; total calories burnt 248–373 kcal; 3 days/wk	↓BM^a^; ↓TC^a^; ↓HDL^a^; ↔ TAG; ↓LDL^a^; ↓V̇O_2max_^a^ (not changed when expressed per kg/bm)
Layman et al. [53]	PG	N = 48, Women Age = 40–56; Overweight/Obese	16 wks	Hypocaloric diet ≈ 1700 kcal	HC + ex (45/15/30); LC + ex (40/30/30),	Walking 5 d/wk & RT 2 d/wk; Walking > 30 min; RT using 7 machines; ≥ 1 set of 12 repetitions per machine to elicit fatigue by the 12th repetition	↓BM^a, e, h^; ↓FM^a, e, h^; ↔ LBM (only in EX groups); ↓in NON-EX; ↓TC^a, e, g^; ↓LDL^a, e, g^; ↓TAG^a^; ↑HDL^c, d^; ↓HDL^b, d^ ↓TAG:HDL^c, d^
Racette et al. [58]	PG	N = 23; Women; Age = 21–47; Overweight/Obese	23 wks	Reduced by 25% from individual’s RMR estimated with the Harris-Bendict formula; ≈1164 kcal/day	Wk 0–5: All groups (45/20/35), Wk 5–17: HC + ex (57/24/19); LC (26/25/49) Wk 17–23: All groups (45/20/35)	3 d/wk; endurance exercise; 45 min/session; 60–65% V̇O_2max_	↓BM^a, d^; ↓FFM^a^; ↓FM^a^; (Aerobic EX had a significant effect in ↓FM *P* < 0.01); ↔ FBG^a^
Ebbeling et al. [56]	CO; 4wks per diet; no washout period	N = 21; Men and Women; Age = 18–40; Overweight/Obese	16 wks	Wk 0–3: hypocaloric for 10–15% weight loss; Wk 4–16: Maintenance diet based on TEE assessed by Stable isotope analysis	Wk 4–16: HC(LF) (59/24/18); LC(VLC) (10/30/60)	Allowed unspecified endurance physical activity but prohibited resistance training	↑HDL^c, f^; ↓HDL^b^; ↓TAG^c, f^; ↓BM^a^; ↑Cortisol^c, e^; ↑Peripheral and hepatic insulin resistance^b, d^
Green D. et al. [51]	CO; 3mo per diet; 2wks washout period	N = 12; Men and Women; Age = 24–53; Normal weight	24 wks	Ad libitum ≈ 2066 kcal	HC (45/22/33); LC (8/23/69)	Competitive Olympic weightlifting training;	↓BM^c, d^; ↓LBM^c, d^; ↔ FM; ↔ 1RM
Das et al. [55]	PG	N = 34; Men and Women; Age = 24–42; Overweight/Obese	48 wks	30% caloric reduction from TEE measured by doubly labeled water [66]	HC (40/30/30); LC (60/20/20)	TDEE measured with Double-Labelled-Water; No training	↓BM^a^; ↓BF^a^; ↓TAG^a^; ↓TC^a^; ↓Insulin^a^; ↑HDL^a^; ↓LDL^a^; ↔ Blood glucose
Volek et al. [36]	CO; 4wks per diet; no reported washout period	N = 26; Women; Age **≈** 25–43; Overweight/Obese	4 wks	− 500 kcal from the nearest 200 kcal increment (≈1931 kcal) of REE obtained using indirect calorimetry; Diet intervention ≈ 1260 kcal/day	HC (59/19/21); LC (9/28/63)	Sedentary/moderately active. Recorded but unspecified	↓TAG:HDL^c, d^; ↓TC^b, d^; ↓LDL^b, d^; ↓HDL^b, d^; ↓VLDL^c, a^; ↓FINS^c, d^; ↓FBG^c, d^; ↓HOMA-IR^c, d^
Keogh et al. [60]	PG	N = 25; Men and Women; Age = 20–65; Obese	12 wks	≈ 1434 kcal to achieve 0.5-1 kg/wk weight loss; ≈ 30% energy restriction based on estimated usual intake using FFQ at baseline [67]	HC (60/20/20); LC (33/40/27)	No training	↓BM^a^; ↓FBG^a^; ↓Insulin^a^; ↓TC^a^; ↔ HDL; ↓LDL^a^; ↓TAG^a^
Bazzano et al. [61]	PG	N = 103; Men and Women; Age = 22–75; Obese	48 wks	Baseline: HC (2034 kcal); LC (1998 kcal) Mo 3: Hypocaloric; HC (1418 kcal); LC (1258 kcal) Mo 6: Hypocaloric; HC (1481 kcal); LC (1324 kcal) Mo 12: Hypocaloric; HC (1527 kcal); LC (1448 kcal)	HC (54/19/30); LC (34/24/41)	Participants in each group were asked to refrain from changing their physical activity levels during the intervention	↓BM^a, e, h^;↓BF^a, d, h^; ↑LBM^c, e^; ↔ TC; ↔ LDL; ↑HDL^c, f^; ↓TC:HDL^c, e^; ↓TAG^c, e^; ↓Insulin^a^; ↔ FBG
Summer et al. [62]	PG	N = 81; Women; Age > 18; Overweight/Obese	16–24 wks	Baseline: HC (1949 kcal); LC (1874 kcal) Post-intervention: HC (1342 kcal); LC (1405 kcal)	HC(AHA Step I) (50/19/31); LC(Atkins) (27/24/49)	Maintained the same level of physical activity. Pedometers were worn to monitor daily step counts	↓BM^c, d, h^; ↓FM^c, d, h^
Morgan et al. [63]	PG	N = 293; Men and Women; Age = 21–60; Overweight/Obese	24 wks	Baseline: HC (2426 kcal); LC (2283 kcal) Mo 2: Hypocaloric; HC (1534 kcal); LC (1627 kcal) Mo 6: Hypocaloric; HC (1485 kcal); LC (1631 kcal)	HC(Rosemary Conley) (48/20/31); LC(Atkins) (18/26/51)	No training	↓BM^a^; ↓LDL^b^; ↓TAG^a^; ↓HDL^b, h^
Buga et al. [47]	CO; 6wks per diet; 2wks washout period	N = 7; Men; Age 18–45; Highly trained endurance athletes	12 wks	Ad libitum; Overal Mean: HC (2837 kcal); LC (2947 kcal)	HC(56/15/28); LC(6/25/69)	Participants were instructed to keep their usual exercise routine unchanged and maintain a consistent training intensity and volume throughout the study	↓TAG^c, e, h^; ↓VLDL^c, e, h^; ↓TAG:HDL^c, e, h^; ↑TC^c, e, h^; ↑LDL^c, e, h^; ↑HDL^c, d, h^ ↔ FBG; ↔ HbA1c

LG: Low glycaemic load; LGI: Low glycaemic index; HG: High glycaemic load; VLC: Very low carbohydrate; CR: Calorie reduction; HC: High carbohydrate; LC: Low carbohydrate; LF: Low fat; wks: weeks; mo: months; PINS: Postprandial insulin; FBG: Fasting blood glucose; BG: Blood glucose; TAG: Triglycerides; PBG: Postprandial blood glucose; FINS: Fasting insulin; TAG: Triglycerides; HDL: High-density lipoprotein; LDL: Low-density lipoprotein; TC: Total Clesterol; WD: Western diet; KD: Ketogenic diet; C: Carbohydrates; P: Protein; F: Fats; PRO: protein group; C: carbohydrate group; EX: Exercise group; LF: Low fat; LBM: Lean body mass; BF: Body fat; BM: Body weight; SQ: Squat; BP: Bench press; RT: Resistance Training; h: Hours; PP: Peak power; PG: Parallel Groups Design; CO: Crossover design

^a^Significant difference p < 0.05 from baseline for both groups; ^b^Significant difference from baseline only for HC; ^c^Significant difference from baseline only for LC; ^d^Significant difference between LC vs HC *P* < 0.05; ^e^significant difference between LC vs HC *P* < 0.01; ^f^: significant difference between LC vs HC *P* < 0.001; ^g^Significantly greater for HC vs LC; ^h^Significantly greater for LC vs HC; ↑: significant increase from baseline; ↓: significant reduction from baseline; ↔ : no significant difference from baseline; ^a^Significantly greater for HC; ^b^Significantly greater for LC; ^c^No difference between HC and LC

### Changes in the analysed variables

#### Primary outcomes

##### Changes in FBG

**Fig. 2:**
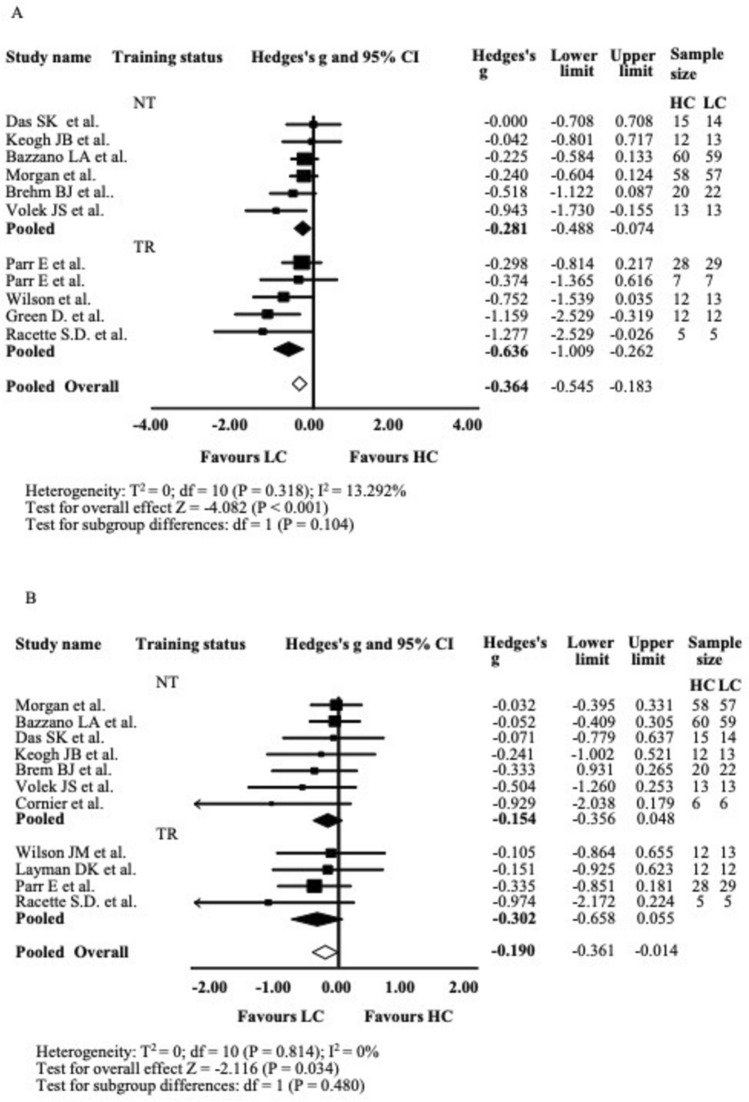
Fast Blood Glucose (FBG), Panel A and Fasting Insulin (FINS), Panel B Forest plot. Results of a random-effects meta-analysis are shown as g effect size with 95% confidence interval. The black and white diamonds represent the subgroups (TR and NT) and pooled (overall) standardised mean difference respectively. TR Training, NT Not-Training, CI confidence interval

From the 11 studies analysing changes in FBG, the mean effect size was small and significant (*g* = − 0.364, 95% CI = − 0.709 to − 0.019, *P* < 0.001). The sensitivity analysis showed that none of the studies contributed disproportionately to the results of the meta-analysis. No significant heterogeneity was found within the 11 treatments. As shown in Fig. 2A, LC diets resulted in greater reductions in fasting blood glucose (FBG) compared to HC diets, with no outliers detected. Among the 11 studies reporting FBG changes, 5 included training (TR) and 6 did not (NT). Both NT and TR subgroups showed significant reductions in fasting blood glucose (NT: *g* = − 0.281, 95% CI = − 0.488 to − 0.074, *P* = 0.008; TR: *g* = − 0.636, 95% CI = − 1.009 to − 0.262, *P* = 0.001). Although the TR subgroup exhibited a larger numerical reduction, the difference in the magnitude of FBG reduction between the TR and NT subgroups was not statistically significant. Funnel plot inspection revealed no major asymmetry, suggesting a low risk of publication bias for this outcome (Fig. S1 supplementary material).

##### Changes in FINS

From the 11 studies analysing changes in FINS, the mean effect size was very small and significant (*g* = − 0.190, 95% CI = − 0.361 to − 0.014, *P* = 0.034). The sensitivity analysis showed that none of the studies contributed disproportionately to the results of the meta-analysis. No significant heterogeneity was found within the 11 treatments. As shown in Fig. 2B, LC diets led to a greater reduction in fasting insulin (FINS) compared to HC diets, with no outliers identified among the included studies. Subgroup analysis of the 11 studies reporting FINS changes revealed that 4 included training (TR) and 7 did not (NT). In the NT subgroup, the effect size was very small and not statistically significant (*g* = − 0.154, 95% CI = − 0.356 to 0.048, *P* = 0.135). Similarly, in the TR subgroup, the effect size was small and non-significant (*g* = − 0.302, 95% CI = − 0.658 to 0.055, *P* = 0.097). No significant differences were found between the TR and NT subgroups in terms of FINS reduction. Visual inspection of the funnel plot showed slight asymmetry, with smaller studies tending toward larger effects, which may indicate mild small-study effects or bias (Fig. S2 supplementary material).

##### Changes in TC

**Fig. 3:**
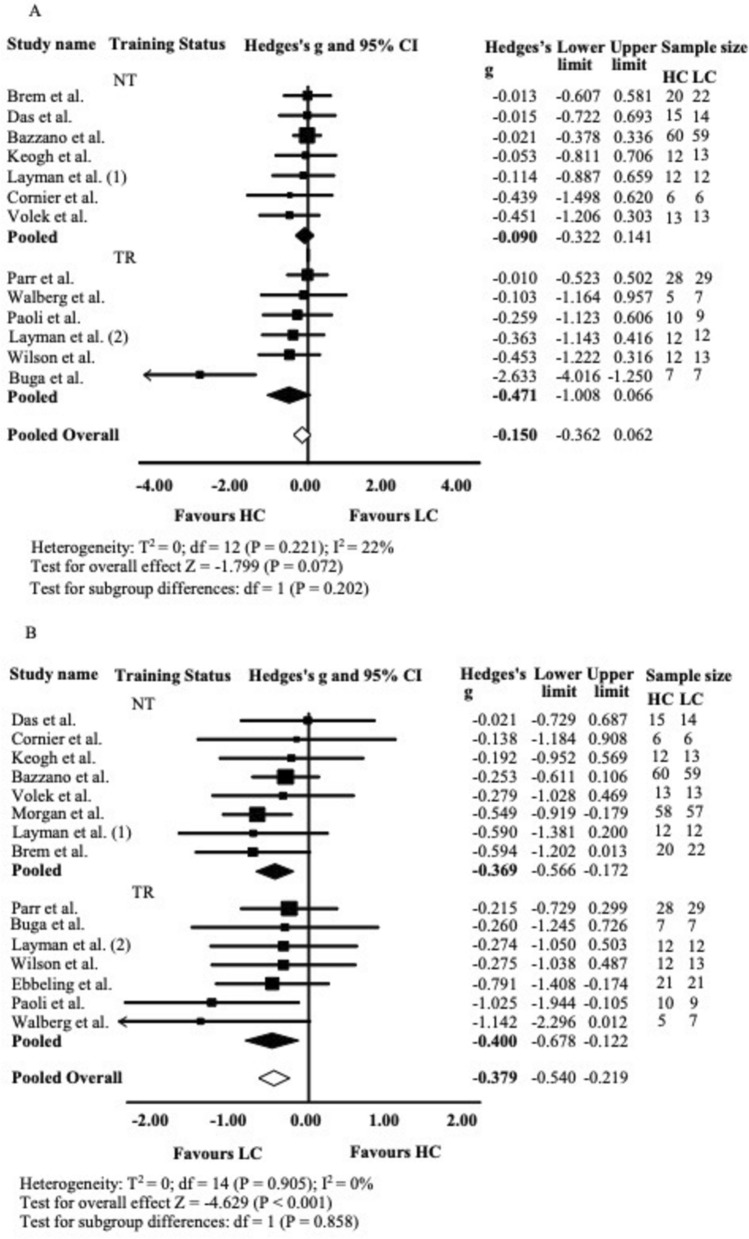
Total Cholesterol (TC), Panel A and Triglycerides (TAG), Panel B Forest plot. Results of a random-effects meta-analysis are shown as g effect size with 95% confidence interval. The black and white diamonds represent the subgroups (TR and NT) and pooled (overall) standardised mean difference respectively. TR Training, NT Not-Training, CI confidence interval

Across 13 studies assessing TC changes, the mean effect size was very small and not statistically significant (*g* = − 0.150; 95% CI − 0.362 to 0.062; *P* = 0.086), with no significant heterogeneity across studies. As shown in Fig. 3A, TC levels did not significantly differ between HC and LC groups. Subgroup analysis of the 13 studies (6 with training [TR], 7 without [NT]) also showed non-significant and small effect sizes for both subgroups (NT, *g* = − 0.090, 95% CI = − 0.322 to 0.141, *P* = 0.443; TR *g* = − 0.471, 95% CI = − 1.008 to 0.066, *P* = 0.086). No significant differences in effect sizes were found between TR and NT subgroups. The funnel plot appeared symmetric, supporting a low likelihood of publication bias for this outcome (Fig. S3 supplementary material).

##### Changes in TAG

From the 15 studies assessing TAG changes, the mean effect size was small to moderate and statistically significant (*g* = − 0.379; 95% CI − 0.540 to − 0.219; *P* < 0.001), with no significant heterogeneity or outliers detected. As shown in Fig. 3B, LC diets reduced TAG more effectively than HC diets. Subgroup analysis (7 TR, 8 NT) revealed significant reductions in both groups, with no significant difference observed between the subgroups (TR: *g* = − 0.400; 95% CI − 0.678 to − 0.122;* P* = 0.005; NT: *g* = − 0.369; 95% CI − 0.566 to − 0.172; *P* < 0.001). The funnel plot showed mild asymmetry favouring LC, which may reflect small-study effects or variability in study size (Fig. S4 supplementary material).

##### Changes in LDL-C

**Fig. 4:**
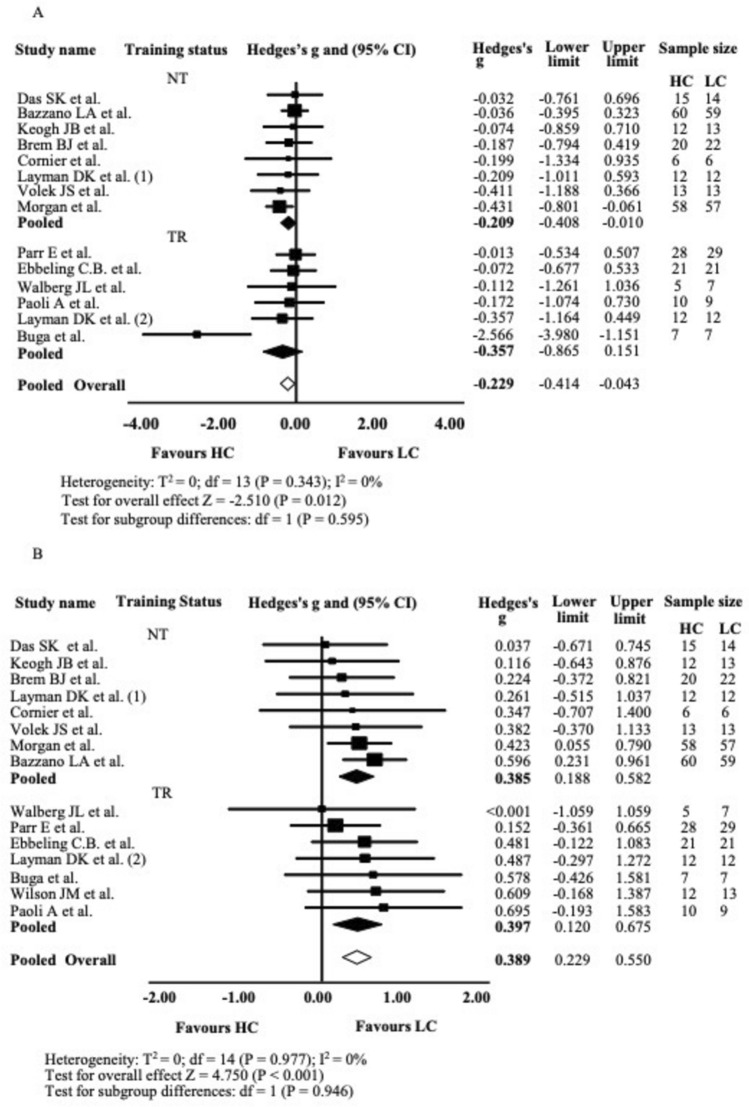
Low-Density Lipoprotein Cholesterol (LDL), Panel A and High-Density Lipoprotein Cholesterol (HDL), Panel B Forest plot. Results of a random-effects meta-analysis are shown as g effect size with 95% confidence interval. The black and white diamonds represent the subgroups (TR and NT) and pooled (overall) standardised mean difference respectively. TR Training, NT Not-Training, CI confidence interval

Across 14 studies assessing LDL-C, the mean effect size was small but statistically significant (*g* = − 0.225; 95% CI − 0.406 to − 0.043; *P* = 0.015), with no significant heterogeneity or outliers identified. As shown in Fig. 4A, HC diets reduced LDL-C more effectively than LC diets. Subgroup analysis (6 TR, 8 NT) showed small, but not significant effects for TR (*g* = − 0.344; 95% CI − 0.831 to 0.143; *P* = 0.166) and small, but significant effects for NT groups (*g* = − 0.205; 95% CI − 0.401 to − 0.010; *P* = 0.040), with no significant difference between subgroups. Funnel plot asymmetry was minimal, indicating a low risk of publication bias in LDL-C comparisons (Fig. S5 supplementary material).

##### Changes in HDL-C

Across 15 studies assessing HDL-C changes, the mean effect size was small to moderate and statistically significant (*g* = 0.389, 95% CI = 0.229 to 0.550, *P* < 0.001), with no evidence of significant heterogeneity or influential outliers. As shown in Fig. 4B, LC diets increased HDL-C significantly more than HC diets. Subgroup analysis (*n* = 15; 7 TR, 8 NT) also revealed significant and similar effects for both TR (*g* = 0.397; 95% CI 0.120 to 0.675;* P* = 0.005) and NT groups (*g* = 0.385; 95% CI 0.188 to 0.582; *P* < 0.001), with no significant differences between subgroups. The HDL-C funnel plot showed mild asymmetry, with some smaller studies reporting larger positive effects, suggesting a possible small-study effect (Fig. S6 supplementary material).

#### Secondary outcomes

##### Changes in BM

From the 11 studies assessing changes in BM, the overall effect size was very small but statistically significant (*g* = − 0.183; 95% CI − 0.349 to − 0.017; *P* = 0.031), with no outliers or significant heterogeneity identified. As shown in Figure S7 (supplementary material), no significant BM differences were found between HC and LC groups. Subgroup analysis (5 TR, 6 NT) also showed minimal effects in both groups (TR: *g* = − 0.154; 95% CI − 0.467 to 0.159; *P* = 0.334; NT: *g* = − 0.195; 95% CI − 0.391 to 0.002; *P* = 0.052), with no significant differences between subgroups. The funnel plot was visually symmetrical, indicating low risk of publication bias for this outcome (Fig. S8 supplementary material).

##### Changes in FM

From the 7 studies assessing FM, the overall effect size was small but significant (*g* = − 0.304; 95% CI − 0.548 to − 0.059; *P* = 0.015), with no outliers or significant heterogeneity detected. As shown in Figure S9 (supplementary material), FM was significantly reduced in the LC groups compared to the HC. Subgroup analysis (5 TR, 2 NT) revealed small, non-significant trends for both groups (NT: *g* = − 0.271; 95% CI − 0.632 to 0.090; *P* = 0.141; TR: *g* = − 0.331; 95% CI − 0.664 to 0.001; *P* = 0.051), with no significant differences between subgroups. Funnel plot inspection suggested some asymmetry, with smaller studies showing stronger LC effects, which may indicate a potential small-study effect (Fig. S10 supplementary material).

##### Changes in FFM

Across 5 studies examining FFM, the overall effect size was very small and not significant (*g* = 0.126; 95% CI − 0.167 to 0.419; *P* = 0.401), with no outliers or significant heterogeneity observed. As shown in Figure S11 (supplementary material), there were no significant differences in FFM between HC and LC groups. Subgroup analysis (3 TR, 2 NT) also showed non-significant and very small effect sizes (TR: *g* = 0.099; 95% CI − 0.275 to 0.473; *P* = 0.602; NT: *g* = 0.168; 95% CI − 0.304 to 0.640; *P* = 0.486), with no differences between subgroups. Although the FFM funnel plot appears visually symmetrical, the number of included studies was small (k = 5). Funnel plot interpretation is unreliable with fewer than 10 studies. Therefore, no conclusions regarding publication bias can be drawn. (Fig. S12 supplementary material).

### Synthesis of results

Following the GRADE approach, the overall quality of evidence was rated as high for the effects of a LC diet (≤ 44% of total daily caloric intake from carbohydrates) compared to a HC diet (≥ 45% CHO). High-certainty evidence supported beneficial effects of the LC diet on HDL-C, TAG, BM, FM, FINS and FBG in healthy adults. Conversely, there was high-certainty evidence favouring the HC diet for reducing LDL-C. The certainty of evidence was rated as low for TC and FFM.

## Discussion

This meta-analysis indicates that, under energy-matched conditions, LC (≤ 44% of TDCI) diets produce modest yet favourable changes in several cardiometabolic health markers compared with HC (≥ 45% TDCI) diets in healthy adults. Variability among studies was mainly attributable to differences in sample size, intervention duration, and methods used to assess energy intake and body composition. These factors were accounted for in the Risk of Bias assessment and are summarised in Supplementary Table S13.

Notably, the effects of both dietary approaches on fasting glucose, HDL-C, triglycerides, LDL-C, body mass, fat mass, and fat-free mass were generally consistent regardless of participants' engagement in regular physical activity. This suggests that the benefits observed are primarily driven by macronutrient composition rather than exercise status.

FBG was significantly reduced in participants following LC diets, with a pooled effect size of *g* = − 0.364. This directionally consistent finding across 8 of 11 studies suggests a robust effect, even in participants with normal glycaemic control at baseline. Although small, this reduction may carry clinical relevance, particularly for individuals at risk of insulin resistance [8]. These effects appear to occur independently of energy restriction, highlighting the role of macronutrient composition in glycaemic regulation [7].

FINS was also lower in the LC groups, with a pooled effect size of *g* = − 0.190, with all the 11 studies showing reductions. These findings align with those of Hashimoto et al. [68], who reported improved insulin sensitivity and lower TAG under energy-matched LC conditions in overweight Japanese men. The trend across studies suggests a beneficial effect of LC diets on early insulin dynamics.

Even though TC showed no significant difference with a pooled effect size of *g* = − 0.150, likely due to offsetting changes in HDL-C and LDL-C, blood lipid responses also favoured LC diets. TAG were significantly reduced with a pooled effect size of *g* = − 0.379 and HDL-C increased significantly with a pooled effect size of *g* = 0.389. These effects were observed across varied populations and durations and align with established physiological responses to reduced carbohydrate intake, including enhanced reverse cholesterol transport and lower hepatic triglyceride synthesis. TAG decreased in 13 of 15 studies, and HDL-C rose in 11 of 15, reinforcing the reliability of these changes. Importantly, the TAG:FBG index (Tyg index) has been strongly correlated with the hyperinsulinemic-euglycemic clamp, the gold standard for assessing insulin sensitivity and has been proven to be better than the HOMA-IR for evaluation of insulin resistance/sensitivity [69–71]. Therefore, the findings that LC diets can decrease insulin resistance are even more relevant as the level of TAG and HDL is directly related to levels of insulin resistance.

Conversely, LDL-C was reduced to a greater extent in HC groups with a pooled effect size of *g* = − 0.225, although this change was small and limited to 7 of 14 studies. Nonetheless, it is worth clarifying that different LDL subfractions have been associated with distinct lipid profiles and varying atherogenic potential. Larger, more buoyant LDL particles (Pattern A) are considered less harmful, whereas smaller, denser LDL particles (Pattern B) remain in circulation for longer periods, are more susceptible to oxidative modification, and more readily penetrate the arterial wall, contributing to atherogenesis. This increased risk is independent of total LDL-C concentration [72]. Moreover, LDL particle size has been shown to increase under LC compared to HC diets, reflecting a shift toward larger, less dense, and less atherogenic LDL subfractions [8, 63]. A recent one-year study of 100 individuals on a ketogenic diet reported substantial elevations in LDL-C and ApoB; however, neither baseline ApoB levels nor their changes were associated with non-calcified plaque volume or total plaque burden, as assessed by coronary computed tomography angiography [73]. Although direct assessment of LDL particle size is not routinely conducted in clinical settings, the TAG to HDL-cholesterol ratio (TAG/HDL-C) is considered a practical surrogate. Ratios exceeding 3.5 are indicative of a predominance of small, dense LDL particles and are frequently associated with insulin resistance [74]. The TAG/HDL-C ratio has been identified as one of the best markers to predict the risk of developing atherosclerosis, cardiovascular disease and type 2 diabetes [74–77]. In addition, a systematic review by Ravnskov et al. [78] challenged this conventional interpretation of LDL-C, reporting no consistent association between LDL-C and cardiovascular disease in individuals over 60 years of age, and even suggested an inverse relationship with all-cause mortality. These findings highlight the complexity of LDL-C as a biomarker and underscore the importance of evaluating lipid profiles within the broader context of age, metabolic status, and particle quality.

In terms of body composition, LC diets led to small but statistically significant reductions in body mass with a pooled effect size of *g* = − 0.183, and FM *g* = − S0.304. No significant differences in FFM were observed with a pooled effect size of *g* = 0.126. The greater reduction of FM in all 12 relevant studies, through the LC, suggests that higher carbohydrate distribution may influence nutrient partitioning, water retention, or satiety, even under energy-matched conditions. The consistent preservation of FFM supports the importance of adequate protein and physical activity over carbohydrate content in maintaining lean tissue [28, 79]. The observed no effect of diet type on FFM reduction also counters common concerns about muscle loss on LC diets when protein intake is ≥ 1.6 g/kg BW and combined with resistance training [50]. These results suggest LC diets may confer modest metabolic advantages in healthy adults without requiring energy restriction. Improvements in FBG, HDL-C, and TAG highlight the role of macronutrient distribution in regulating cardiometabolic risk.

Our findings support previous meta-analyses, including those by Santos et al. [80] and Mansoor et al. [81], which also reported improved glycaemic and lipid outcomes with LC diets. However, our review differs in its exclusive focus on energy-matched interventions in healthy adult populations. Prior reviews included trials with hypocaloric protocols, pharmacological interventions, or participants with obesity or metabolic disease and adolescents [68, 82–85]. By isolating the effect of carbohydrate distribution under energy-controlled conditions, the current review offers a clearer understanding of its independent impact.

Additionally, consistent findings across studies with varied participant sex, training status, and intervention durations suggest that the benefits of LC diets may extend to a broad range of healthy individuals. The small effect sizes (especially in glycaemic outcomes) reflect the already healthy baseline profiles of the participants, which may have limited the degree of observable change.

In summary, LC diets resulted in more favourable changes in fasting glucose, HDL-C, and TAG compared to HC diets under energy-matched conditions. Reductions in LDL-C were slightly more pronounced with HC diets, though the broader lipid profile supported LC benefits. Favourable reductions in body mass and FM occurred with LC diets, while FFM was preserved. These findings indicate that LC diets (< 45% TDCI) are a viable dietary strategy for improving cardiometabolic and body composition markers in healthy adults.

Although the present analysis focused on directly measured outcomes such as glycaemic and lipid markers, LC and HC diets may also exert broader metabolic effects through hormonal and molecular pathways [37, 86, 87]. Factors such as insulin, leptin [88, 89], cortisol, and sex hormones [90–101] could partly mediate the observed differences, warranting further mechanistic research beyond the scope of this meta-analysis.

The differential effects observed between LC and HC diets may partly reflect hormonal adaptations influencing substrate utilization and energy partitioning. Lower carbohydrate intake can reduce postprandial insulin secretion, thereby promoting greater reliance on lipid oxidation and improving triglyceride clearance. Conversely, higher carbohydrate intake increases hepatic glycogen storage and may enhance LDL-cholesterol clearance through upregulation of LDL receptors. These mechanisms, together with differences in leptin and cortisol responses, may underlie the observed lipid and glycaemic patterns.

### Limitations and recommendations for future studies

Several limitations should be considered when interpreting these results. Although most of the included studies lasted more than eight weeks, the minimum threshold of four weeks may not fully capture longer-term physiological adaptations. Additionally, all trials were conducted under free-living conditions, limiting dietary control. Despite the use of food diaries, consultations, and biochemical monitoring, self-reported dietary intake is subject to underreporting and measurement error [102–105].

To target healthy, non-medicated populations, studies containing the terms ‘patient’ or ‘hospit*’* were intentionally excluded; however, this restriction may have inadvertently omitted some eligible trials involving clinically stable individuals, constituting a minor limitation.

There was considerable heterogeneity in participant characteristics, including sex, physical activity levels, and body composition. While all participants were classified as healthy adults, such variation may reduce the generalisability of the pooled estimates. Sample sizes also varied widely, influencing the weighting and precision of individual study outcomes. Additionally, hormonal profiles and menopausal status were rarely reported, despite their known influence on lipid metabolism and insulin sensitivity.

Future studies should aim to implement longer interventions, controlled feeding environments, and more homogeneous participant selection. Stratified reporting by sex, hormonal status, and physical activity level would allow for more nuanced interpretation and enhance the accuracy and applicability of findings.

## Conclusions

LC diets (≤ 44% TDCI) result in modest but statistically significant improvements in HDL-C, TAG, FBG, BM, and FINS compared to HC diets (≥ 45% TDCI) under energy-matched conditions in healthy adults. Importantly, these effects were largely consistent in both physically active and inactive individuals. In contrast, HC diets were more effective in reducing LDL-C. No significant differences were observed in TC and FFM. These findings support the role of carbohydrate distribution, independent of physical activity status, in shaping metabolic and body composition outcomes.

## Supplementary Information

Below is the link to the electronic supplementary material.

Supplementary file1 (DOCX 42 KB)

Supplementary file2 (DOCX 30 KB)

Supplementary file3 (DOCX 29 KB)

Supplementary file4 (PDF 56 KB)

Supplementary file5 (PDF 55 KB)

Supplementary file6 (PDF 55 KB)

Supplementary file7 (PDF 56 KB)

Supplementary file8 (PDF 55 KB)

Supplementary file9 (PDF 56 KB)

Supplementary file10 (JPG 140 KB)

Supplementary file11 (PDF 54 KB)

Supplementary file12 (JPG 118 KB)

Supplementary file13 (PDF 53 KB)

Supplementary file14 (JPG 105 KB)

Supplementary file15 (PDF 52 KB)

## Data Availability

Data will be provided on request.

## References

[1] Lobstein T, Jackson-Leach R, Powis J et al (2023) World atlas obesity. World Obesity Federation, London

[2] Emmerich SD, Fryar CD, Stierman B, Ogden CL (2021) Obesity and severe obesity prevalence in adults: United States, August 2021-August 2023 Key findings Data from the National Health and Nutrition Examination Survey 10.15620/cdc/159281 PMC11744423 39808758

[3] Janssen JAMJL (2022) New insights into the role of insulin and hypothalamic-pituitary-adrenal (HPA) axis in the metabolic syndrome. Int J Mol Sci 23(15):8178 35897752 10.3390/ijms23158178 PMC9331414

[4] Taylor R, Ramachandran A, Yancy WS, Forouhi NG (2021) Nutritional basis of type 2 diabetes remission. The BMJ. 10.1136/bmj.n1449 34233884 10.1136/bmj.n1449 PMC8261662

[5] Kolb H, Kempf K, Röhling M, Martin S (2020) Insulin: too much of a good thing is bad. BMC Med. 10.1186/s12916-020-01688-6 32819363 10.1186/s12916-020-01688-6 PMC7441661

[6] Kolb H, Stumvoll M, Kramer W et al (2018) Insulin translates unfavourable lifestyle into obesity. BMC Med. 10.1186/s12916-018-1225-1 30541568 10.1186/s12916-018-1225-1 PMC6292073

[7] Astley CM, Todd JN, Salem RM et al (2018) Genetic evidence that carbohydrate-stimulated insulin secretion leads to obesity. Clin Chem 64:192–200. 10.1373/clinchem.2017.280727 29295838 10.1373/clinchem.2017.280727 PMC5937525

[8] Siri-Tarino PW, Sun Q, Hu FB, Krauss RM (2010) Saturated fat, carbohydrate, and cardiovascular disease. Am J Clin Nutr 91:502–509 20089734 10.3945/ajcn.2008.26285 PMC2824150

[9] Malhotra A, Redberg RF, Meier P (2017) Saturated fat does not clog the arteries: coronary heart disease is a chronic inflammatory condition, the risk of which can be effectively reduced from healthy lifestyle interventions. Br J Sports Med 51:1111–1112 28442474 10.1136/bjsports-2016-097285

[10] Teicholz N (2023) A short history of saturated fat: the making and unmaking of a scientific consensus. Curr Opin Endocrinol Diabetes Obes 30:65–71 36477384 10.1097/MED.0000000000000791 PMC9794145

[11] Kang ZQ, Yang Y, Xiao B (2020) Dietary saturated fat intake and risk of stroke: systematic review and dose–response meta-analysis of prospective cohort studies. Nutr Metab Cardiovasc Dis. 10.1016/j.numecd.2019.09.028 31791641 10.1016/j.numecd.2019.09.028

[12] GrundyScott M, HBBJICSCSCL (2004) Definition of metabolic syndrome: report of the National Heart, Lung, and Blood Institute/American Heart Association conference on scientific issues related to definition. Circulation 109:433–438. 10.1161/01.CIR.0000111245.75752.C6 14744958 10.1161/01.CIR.0000111245.75752.C6

[13] Paoli A, Rubini A, Volek JS, Grimaldi KA (2013) Beyond weight loss: a review of the therapeutic uses of very-low-carbohydrate (ketogenic) diets. Eur J Clin Nutr 67:789–796. 10.1038/ejcn.2013.116 23801097 10.1038/ejcn.2013.116 PMC3826507

[14] Gardner CD, Kiazand A, Alhassan S et al (2007) Comparison of the Atkins, Zone, Ornish, and LEARN diets for change in weight and related risk factors among overweight premenopausal women the A TO Z weight loss study: a randomized trial. JAMA 297(9):969–977. 10.1001/jama.297.9.969 17341711 10.1001/jama.297.9.969

[15] Volek JS, Phinney SD, Forsythe CE et al (2009) Carbohydrate restriction has a more favorable impact on the metabolic syndrome than a low fat diet. Lipids 44:297–309. 10.1007/s11745-008-3274-2 19082851 10.1007/s11745-008-3274-2

[16] Johnston CS, Tjonn SL, Swan PD et al (2006) Ketogenic low-carbohydrate diets have no metabolic advantage over nonketogenic low-carbohydrate diets. Am J Clin Nutr 2006(5):1055–1061. 10.1093/ajcn/83.5.1055 10.1093/ajcn/83.5.1055 16685046

[17] Sharman MJ, Kraemer WJ, Love DM et al (2002) Human nutrition and metabolism a ketogenic diet favorably affects serum biomarkers for cardiovascular disease in normal-weight men. J Nutr 132(7):1879–1885. 10.1093/jn/132.7.1879 12097663 10.1093/jn/132.7.1879

[18] Gardner CD, Trepanowski JF, Gobbo LCD et al (2018) Effect of low-fat VS low-carbohydrate diet on 12-month weight loss in overweight adults and the association with genotype pattern or insulin secretion the DIETFITS randomized clinical trial. JAMA 319:667–679. 10.1001/jama.2018.0245 29466592 10.1001/jama.2018.0245 PMC5839290

[19] Vargas-Molina S, Carbone L, Romance R et al (2021) Effects of a low-carbohydrate ketogenic diet on health parameters in resistance-trained women. Eur J Appl Physiol 121:2349–2359. 10.1007/s00421-021-04707-3 34003364 10.1007/s00421-021-04707-3

[20] Smart NA, Downes D, van der Touw T et al (2024) The effect of exercise training on blood lipids: a systematic review and meta-analysis. Sports Med. 10.1007/s40279-024-02115-z 39331324 10.1007/s40279-024-02115-z PMC11787149

[21] Sampath Kumar A, Maiya AG, Shastry BA et al (2019) Exercise and insulin resistance in type 2 diabetes mellitus: a systematic review and meta-analysis. Ann Phys Rehabil Med 62:98–103 30553010 10.1016/j.rehab.2018.11.001

[22] Zhang H, Guo Y, Hua G et al (2024) Exercise training modalities in prediabetes: a systematic review and network meta-analysis. Front Endocrinol (Lausanne) 15:1308959 38440785 10.3389/fendo.2024.1308959 PMC10911289

[23] Ryan BJ, Schleh MW, Ahn C et al (2020) Moderate-intensity exercise and high-intensity interval training affect insulin sensitivity similarly in obese adults. J Clin Endocrinol Metab 105:E2941–E2959. 10.1210/clinem/dgaa345 32492705 10.1210/clinem/dgaa345 PMC7347288

[24] Almuraikhy S, Doudin A, Domling A et al (2024) Molecular regulators of exercise-mediated insulin sensitivity in non-obese individuals. J Cell Mol Med. 10.1111/jcmm.18015 37938877 10.1111/jcmm.18015 PMC10805515

[25] Conn VS, Koopman RJ, Ruppar TM et al (2014) Insulin sensitivity following exercise interventions: systematic review and meta-analysis of outcomes among healthy adults. J Prim Care Community Health 5:211–222 24474665 10.1177/2150131913520328 PMC4393364

[26] Public Health England (2018) The Eatwell Guide. Public Health England

[27] Dietary Guidelines for Americans-2020–2025 10.1177/21650799211026980 34279148

[28] Aragon AA, Schoenfeld BJ, Wildman R et al (2017) International society of sports nutrition position stand: diets and body composition. J Int Soc Sports Nutr. 10.1186/s12970-017-0174-y 28630601 10.1186/s12970-017-0174-y PMC5470183

[29] Liberati A, Altman DG, Tetzlaff J et al (2009) The PRISMA statement for reporting systematic reviews and meta-analyses of studies that evaluate health care interventions: explanation and elaboration. PLoS Med 6(7):e1000100. 10.1371/journal.pmed.1000100 19621070 10.1371/journal.pmed.1000100 PMC2707010

[30] Page MJ, McKenzie JE, Bossuyt PM et al (2021) The PRISMA 2020 statement: An updated guideline for reporting systematic reviews. The BMJ. 10.1136/bmj.n71 33782057 10.1136/bmj.n71 PMC8005924

[31] Shamseer L, Moher D, Clarke M et al (2015) Preferred reporting items for systematic review and meta-analysis protocols (prisma-p) 2015: elaboration and explanation. BMJ 350:g7647. 10.1136/bmj.g7647 25555855 10.1136/bmj.g7647

[32] Moher D, Tricco AC (2008) Issues related to the conduct of systematic reviews: a focus on the nutrition field. Am J Clin Nutr 88:1191–1199 18996852 10.3945/ajcn.2008.26255

[33] Johnston CS, Tjonn SL, Swan PD (2004) High-protein, low-fat diets are effective for weight loss and favorably alter biomarkers in healthy adults. J Nutr 2004:586–591. 10.1093/jn/134.3.586 10.1093/jn/134.3.586 14988451

[34] Sun J, Ruan Y, Xu N et al (2023) The effect of dietary carbohydrate and calorie restriction on weight and metabolic health in overweight/obese individuals: a multi-center randomized controlled trial. BMC Med 21:28. 10.1186/s12916-023-02869-9 37226271 10.1186/s12916-023-02869-9 PMC10210464

[35] McAuley KA, Hopkins CM, Smith KJ et al (2005) Comparison of high-fat and high-protein diets with a high-carbohydrate diet in insulin-resistant obese women. Diabetologia 48:8–16. 10.1007/s00125-004-1603-4 15616799 10.1007/s00125-004-1603-4

[36] Volek JS, Sharman MJ, Gómez AL et al (2004) Comparison of a very low-carbohydrate and low-fat diet on fasting lipids, LDL subclasses, insulin resistance, and postprandial lipemic responses in overweight women. J Am Coll Nutr 23:177–184. 10.1080/07315724.2004.10719359 15047685 10.1080/07315724.2004.10719359

[37] Cornier M-A, Troy Donahoo W, Pereira R et al (2005) Human physiology insulin sensitivity determines the effectiveness of dietary macronutrient composition on weight loss in obese women. Obes Res 13(4):703–709. 10.1038/oby.2005.79 15897479 10.1038/oby.2005.79

[38] Grundy SM, Cleeman JI, Daniels SR et al (2005) Diagnosis and management of the metabolic syndrome: an American Heart Association/National Heart, Lung, and Blood Institute scientific statement: executive summary. Crit Pathw Cardiol 4:198–203. 10.1097/00132577-200512000-00018 18340209 10.1097/00132577-200512000-00018

[39] Trumbo P, Schlicker S, Yates AA, Poos M (2002) Dietary reference intakes for energy, carbohydrate, fiber, fat, fatty acids, cholesterol, protein and amino acids. J Am Diet Assoc 102:1621–1630. 10.1016/s0002-8223(02)90346-9 12449285 10.1016/s0002-8223(02)90346-9

[40] Gardner CD, Vadiveloo MK, Petersen KS et al (2023) Popular dietary patterns: alignment with American Heart Association 2021 dietary guidance: a scientific statement from the American Heart Association. Circulation 147:1715–1730 37128940 10.1161/CIR.0000000000001146

[41] Layman DK, Evans EM, Erickson D et al (2009) A moderate-protein diet produces sustained weight loss and long-term changes in body composition and blood lipids in obese adults. J Nutr 139:514–521. 10.3945/jn.108.099440 19158228 10.3945/jn.108.099440

[42] Sharman MJ, Gómez AL, Kraemer WJ, Volek JS (2004) Very low-carbohydrate and low-fat diets affect fasting lipids and postprandial lipemia differently in overweight men. J Nutr 134(4):880–885. 10.1093/jn/134.4.880 15051841 10.1093/jn/134.4.880

[43] Parr EB, Coffey VG, Cato LE et al (2016) A randomized trial of high-dairy-protein, variable-carbohydrate diets and exercise on body composition in adults with obesity. Obesity 24:1035–1045. 10.1002/oby.21451 26931302 10.1002/oby.21451

[44] Heilbronn LK, De Jonge L, Frisard MI et al (2006) Effect of 6-month calorie restriction on biomarkers of longevity, metabolic adaptation, and oxidative stress in overweight individuals a randomized controlled trial. JAMA 295(13):1539–1548. 10.1001/jama.295.13.1539 16595757 10.1001/jama.295.13.1539 PMC2692623

[45] Lin S, Cienfuegos S, Ezpeleta M et al (2023) Effect of time-restricted eating versus daily calorie restriction on mood and quality of life in adults with obesity. Nutrients. 10.3390/nu15204313 37892388 10.3390/nu15204313 PMC10609268

[46] Crabtree CD, Kackley ML, Buga A, Fell B, LaFountain RA, Hyde PN, Sapper TN, Kraemer WJ, Scandling D, Simonetti OP et al (2021) Comparison of ketogenic diets with and without ketone salts versus a low-fat diet: liver fat responses in overweight adults. Nutrients 13(3):966. 10.3390/nu13030966 33802651 10.3390/nu13030966 PMC8002465

[47] Buga A, Buga CM, Ding H et al (2022) Effects of ketogenic diets with or without exogenous ketone salts on cardiometabolic health in adults with overweight: a randomized controlled trial. Nutrients 14(11):1135. 10.3390/nu1410113535334791

[48] Sterne JAC, Savović J, Page MJ et al (2019) RoB 2: a revised tool for assessing risk of bias in randomised trials. The BMJ. 10.1136/bmj.l4898 31462531 10.1136/bmj.l4898

[49] Apong PE (2019) Nutrition and dietary recommendations for bodybuilders. In: Bagchi D, Nair S, Sen CK (eds) Nutrition and enhanced sports performance: muscle building, endurance, and strength, 2nd edn. Elsevier Academic Press, Netherland, pp 737–750

[50] Wilson JM, Lowery RP, Roberts MD et al (2020) Effects of ketogenic dieting on body composition, strength, power, and hormonal profiles in resistance training men. J Strength Cond Res 34(12):3463–3474. 10.1519/JSC.0000000000001935 28399015 10.1519/JSC.0000000000001935

[51] Greene DA, Varley BJ, Hartwig TB et al (2018) A low-carbohydrate ketogenic diet reduces body mass without compromising performance in powerlifting and Olympic weightlifting athletes. J Strength Cond Res 32(12):3373–3382. 10.1519/JSC.0000000000002904 30335720 10.1519/JSC.0000000000002904

[52] Paoli A, Cenci L, Pompei PL et al (2021) Effects of two months of very low carbohydrate ketogenic diet on body composition, muscle strength, muscle area, and blood parameters in competitive natural body builders. Nutrients 13:1–14. 10.3390/nu13020374 10.3390/nu13020374 PMC7911670 33530512

[53] Layman DK, Evans E, Baum JI et al (2005) Human nutrition and metabolism dietary protein and exercise have additive effects on body composition during weight loss in adult women. J Nutr 135(8):1903–1910. 10.1093/jn/135.8.1903 16046715 10.1093/jn/135.8.1903

[54] Layman DK, Boileau RA, Erickson DJ et al (2003) A reduced ratio of dietary carbohydrate to protein improves body composition and blood lipid profiles during weight loss in adult women. J Nutr 133(2):411–417. 10.1093/jn/133.2.411 12566476 10.1093/jn/133.2.411

[55] Krupa Das S, Gilhooly CH, Golden JK et al (2007) Long-term effects of 2 energy-restricted diets differing in glycemic load on dietary adherence, body composition, and metabolism in CALERIE: a 1-y randomized controlled trial. Am J Clin Nutr 85(4):1023–1030. 10.1093/ajcn/85.4.1023 17413101 10.1093/ajcn/85.4.1023

[56] Ebbeling CB, Swain JF, Feldman HA et al (2012) Effects of dietary composition on energy expenditure during weight-loss maintenance. JAMA 307:2627–2634. 10.1001/jama.2012.6607 22735432 10.1001/jama.2012.6607 PMC3564212

[57] Brehm BJ, Seeley RJ, Daniels SR, D’Alessio DA (2003) A randomized trial comparing a very low carbohydrate diet and a calorie-restricted low fat diet on body weight and cardiovascular risk factors in healthy women. J Clin Endocrinol Metab 88:1617–1623. 10.1210/jc.2002-021480 12679447 10.1210/jc.2002-021480

[58] Racette SB, Schoeller DA, Kushner RF et al (1995) Effects of aerobic exercise and dietary carbohydrate on energy expenditure and body composition during weight reduction in obese women. Am J Clin Nutr 1995:486–494. 10.1093/ajcn/61.3.486 10.1093/ajcn/61.3.486 7872211

[59] Walberg JL, Ruiz VK, Tarlton SL et al (1988) Exercise capacity and nitrogen loss during a high or low carbohydrate diet. Med Sci Sports Exerc 20:34–43. 10.1249/00005768-198802000-00005 3343914 10.1249/00005768-198802000-00005

[60] Keogh JB, Brinkworth GD, Clifton PM (2007) Effects of weight loss on a low-carbohydrate diet on flow-mediated dilatation, adhesion molecules and adiponectin. Br J Nutr 98:852–859. 10.1017/S0007114507747815 17490508 10.1017/S0007114507747815

[61] Bazzano LA, Hu T, Reynolds K et al (2014) Effects of low-carbohydrate and low-fat diets: a randomized trial. Ann Intern Med 161:309–318. 10.7326/M14-0180 25178568 10.7326/M14-0180 PMC4428290

[62] Summer SS, Brehm BJ, Benoit SC, D’Alessio DA (2011) Adiponectin changes in relation to the macronutrient composition of a weight-loss diet. Obesity 19:2198–2204. 10.1038/oby.2011.60 21455123 10.1038/oby.2011.60

[63] Morgan L, Griffin B, Millward D et al (2009) Comparison of the effects of four commercially available weight-loss programmes on lipid-based cardiovascular risk factors. Public Health Nutr 12:799–807. 10.1017/S1368980008003236 18647427 10.1017/S1368980008003236

[64] Volek JS, Sharman MJ (2004) Cardiovascular and hormonal aspects of very-low-carbohydrate ketogenic diets. Obes Res 12(Suppl 2):115S-S123. 10.1038/oby.2004.276 15601959 10.1038/oby.2004.276

[65] Frankenfield D, Roth-Yousey L, Compher C (2005) Comparison of predictive equations for resting metabolic rate in healthy nonobese and obese adults: a systematic review. J Am Diet Assoc 105(5):775–789. 10.1016/j.jada.2005.02.005 15883556 10.1016/j.jada.2005.02.005

[66] Schoeller DA (1988) Measurement of energy expenditure in free-living humans by using doubly labeled water. J Nutr 118:1278–1289. 10.1093/jn/118.11.1278 3142975 10.1093/jn/118.11.1278

[67] Hodge A, Patterson AJ, Brown WJ, Ireland P, Giles G (2000) The anti-cancer council of Victoria FFQ: relative validity of nutrient intakes compared with weighed food records in young to middle-aged women in a study of iron supplementation. Aust N Z J Public Health 24(6):576–583. 10.1111/j.1467-842X.2000.tb00520.x 11215004 10.1111/j.1467-842x.2000.tb00520.x

[68] Hashimoto Y, Fukuda T, Oyabu C et al (2016) Impact of low-carbohydrate diet on body composition: meta-analysis of randomized controlled studies. Obes Rev 17:499–509. 10.1111/obr.12405 27059106 10.1111/obr.12405

[69] Guerrero-Romero F, Simental-Mendía LE, González-Ortiz M et al (2010) The product of triglycerides and glucose, a simple measure of insulin sensitivity. Comparison with the euglycemic-hyperinsulinemic clamp. J Clin Endocrinol Metab 95:3347–3351. 10.1210/jc.2010-0288 20484475 10.1210/jc.2010-0288

[70] Luo P, Cao Y, Li P, et al (2022) TyG index performs better than HOMA-IR in Chinese type 2 diabetes mellitus with a BMI <35 kg/m^2^: a hyperglycemic clamp validated study. Med (Lithuania) 58(7):876. 10.3390/medicina58070876 10.3390/medicina58070876 PMC9325243 35888595

[71] Chen X, Liu D, He W et al (2023) Predictive performance of triglyceride glucose index (TyG index) to identify glucose status conversion: a 5-year longitudinal cohort study in Chinese pre-diabetes people. J Transl Med. 10.1186/s12967-023-04402-1 37715242 10.1186/s12967-023-04402-1 PMC10503019

[72] Howard BV, Van Horn L, Hsia J et al (2006) Low-fat dietary pattern and risk of cardiovascular disease: the Women’s Health Initiative randomized controlled dietary modification trial. JAMA 295:655–666. 10.1001/jama.295.6.655 16467234 10.1001/jama.295.6.655

[73] Soto-Mota A, Norwitz NG, Manubolu VS et al (2025) Plaque Begets Plaque, ApoB does not longitudinal data from the KETO-CTA Trial. 10.1016/j.jacadv.2025.101686 10.1016/j.jacadv.2025.101686 PMC12418260 40192608

[74] McLaughlin T, Reaven G, Abbasi F et al (2005) Is there a simple way to identify insulin-resistant individuals at increased risk of cardiovascular disease? Am J Cardiol 96:399–404. 10.1016/j.amjcard.2005.03.085 16054467 10.1016/j.amjcard.2005.03.085

[75] Li Y, Shi H, Liang C et al (2024) The relationship between triglyceride to high-density lipoprotein cholesterol ratio and cardiovascular high-risk: a cross-sectional investigation. 10.1101/2024.05.08.24307048 10.3389/fendo.2025.1688624 PMC12695605 41393287

[76] Yuge H, Okada H, Hamaguchi M et al (2023) Triglycerides/HDL cholesterol ratio and type 2 diabetes incidence: Panasonic Cohort Study 10. Cardiovasc Diabetol. 10.1186/s12933-023-02046-5 37940952 10.1186/s12933-023-02046-5 PMC10634002

[77] Volek JS, Feinman RD (2005) Carbohydrate restriction improves the features of metabolic syndrome. Metabolic syndrome may be defined by the response to carbohydrate restriction. Nutr Metab Lond 2:1–17. 10.1186/1743-7075-2-31 16288655 10.1186/1743-7075-2-31 PMC1323303

[78] Ravnskov U, Diamond DM, Hama R et al (2016) Lack of an association or an inverse association between low-density-lipoprotein cholesterol and mortality in the elderly: a systematic review. BMJ Open. 10.1136/bmjopen-2015-010401 27292972 10.1136/bmjopen-2015-010401 PMC4908872

[79] Stokes T, Hector AJ, Morton RW et al (2018) Recent perspectives regarding the role of dietary protein for the promotion of muscle hypertrophy with resistance exercise training. Nutrients 10(2):180. 10.3390/nu10020180 29414855 10.3390/nu10020180 PMC5852756

[80] Santos FL, Esteves SS, da Costa Pereira A et al (2012) Systematic review and meta-analysis of clinical trials of the effects of low carbohydrate diets on cardiovascular risk factors. Obes Rev 13:1048–1066. 10.1111/j.1467-789X.2012.01021.x 22905670 10.1111/j.1467-789X.2012.01021.x

[81] Mansoor N, Vinknes KJ, Veierod MB, Retterstol K (2016) Effects of low-carbohydrate diets v. low-fat diets on body weight and cardiovascular risk factors a meta-analysis of randomised controlled trials. Br J Nutr 115:466–479. 10.1017/S0007114515004699 26768850 10.1017/S0007114515004699

[82] Bravata DM, Sanders L, Huang J et al (2003) Efficacy and safety of low-carbohydrate diets a systematic review. JAMA 289(14):1837–1850. 10.1001/jama.289.14.1837 12684364 10.1001/jama.289.14.1837

[83] Nordmann AJ, Nordmann A, Briel M et al (2006) Effects of low-carbohydrate vs low-fat diets on weight loss and cardiovascular risk factors. Arch Intern Med 166(3):285–293. 10.1001/archinte.166.3.285 16476868 10.1001/archinte.166.3.285

[84] Hession M, Rolland C, Kulkarni U et al (2009) Systematic review of randomized controlled trials of low-carbohydrate vs. low-fat/low-calorie diets in the management of obesity and its comorbidities. Obes Rev 10(1):36–50. 10.1111/j.1467-789X.2008.00518.x 18700873 10.1111/j.1467-789X.2008.00518.x

[85] Hu T, Mills KT, Yao L et al (2012) Effects of low-carbohydrate diets versus low-fat diets on metabolic risk factors: a meta-analysis of randomized controlled clinical trials. Am J Epidemiol 176(Suppl 7):S44–S54. 10.1093/aje/kws264 23035144 10.1093/aje/kws264 PMC3530364

[86] Cederberg A, Grønning LM, Ahren B et al (2001) FOXC2 is a winged helix gene that counteracts obesity, hypertriglyceridemia, and diet-induced insulin resistance. Cell 106(5):563–573. 10.1016/S0092-8674(01)00474-3 11551504 10.1016/s0092-8674(01)00474-3

[87] Wang Y, Hua S, Cui X et al (2020) The effect of FOXC2-AS1 on white adipocyte browning and the possible regulatory mechanism. Front Endocrinol (Lausanne) 11:565483. 10.3389/fendo.2020.565483 33193083 10.3389/fendo.2020.565483 PMC7658007

[88] Jenkins AB, Markovic TP, Fleury A, Campbell LV (1997) Carbohydrate intake and short-term regulation of leptin in humans. Diabetologia 40:348–351. 10.1007/s001250050851 9084976 10.1007/s001250050686

[89] Izadi V, Saraf-Bank S, Azadbakht L (2014) Dietary intakes and leptin concentrations. ARYA Atheroscler 10(5):266–272 25477984 PMC4251481

[90] Kraemer WJ (1988) Endocrine responses to resistance exercise. Med Sci Sports Exerc 20(5 Suppl):S152–S157. 10.1249/00005768-198810001-00011 3057315 10.1249/00005768-198810001-00011

[91] Bélanger A, Locong A, Noël C et al (1989) Influence of diet on plasma steroids and sex hormone-binding globulin levels in adult men. J Steroid Biochem 32(6):829–833. 10.1016/0022-4731(89)90459-7 2526906 10.1016/0022-4731(89)90459-7

[92] Goldin BR, Woods MN, Spiegelman DL et al (1994) The effect of dietary fat and fiber on serum estrogen concentrations in premenopausal women under controlled dietary conditions. Cancer 74(3 Suppl):1125–1131. 10.1002/1097-0142(19940801)74:3+%3c1125:AID-CNCR2820741521%3e3.0.CO;2-5 8039147 10.1002/1097-0142(19940801)74:3+<1125::aid-cncr2820741521>3.0.co;2-5

[93] Volek JS, Kraemer WJ, Bush JA, Incledon T, Boetes M (1997) Testosterone and cortisol in relationship to dietary nutrients and resistance exercise. J Appl Physiol 82(1):49–54. 10.1152/jappl.1997.82.1.49 9029197 10.1152/jappl.1997.82.1.49

[94] Hämäläinen E, Adlercreutz H, Puska P et al (1984) Diet and serum sex hormones in healthy men. J Steroid Biochem 20(1):459–464. 10.1016/0022-4731(84)90254-1 6538617 10.1016/0022-4731(84)90254-1

[95] Hill PB, Wynder EL (1979) Effect of a vegetarian diet and dexamethasone on plasma prolactin, testosterone and dehydroepiandrosterone in men and women. Cancer Lett 7(5):273–282. 10.1016/S0304-3835(79)80054-3 159772 10.1016/s0304-3835(79)80054-3

[96] Howie BJ, Donaldson DS (1985) Dietary and hormonal interrelationships among vegetarian Seventh-Day Adventists and nonvegetarian men. Am J Clin Nutr 42(1):127–134. 10.1093/ajcn/42.1.127 4014062 10.1093/ajcn/42.1.127

[97] Ingram D, Brown W, Curnow C et al (1987) Effect of low-fat diet on female sex hormone levels. J Natl Cancer Inst 79(6):1225–1229 3480374

[98] Key TJ, Roe L, Thorogood M et al (1990) Testosterone, sex hormone-binding globulin, calculated free testosterone, and oestradiol in male vegans and omnivores. Br J Nutr 64:111–119. 10.1079/BJN19900014 2400756 10.1079/bjn19900014

[99] Raben A, Kiens B, Richter EA et al (1992) Serum sex hormones and endurance performance after a lacto-ovo vegetarian and a mixed diet. Med Sci Sports Exerc 24(11):1290–1297. 10.1249/00005768-199211000-00015 1435181

[100] Reed MJ, Cheng RW, Simmonds M, Richmond W, James VHT (1987) Dietary lipids: an additional regulator of plasma levels of sex hormone-binding globulin. J Clin Endocrinol Metab 64(5):1083–1085. 10.1210/jcem-64-5-1083 3558725 10.1210/jcem-64-5-1083

[101] Morisset AS, Blouin K, Tchernof A (2008) Impact of diet and adiposity on circulating levels of sex hormone-binding globulin and androgens. Nutr Rev 66(9):506–516. 10.1111/j.1753-4887.2008.00083.x 18752474 10.1111/j.1753-4887.2008.00083.x

[102] Foster E, Lee C, Imamura F et al (2019) Validity and reliability of an online self-report 24-h dietary recall method (Intake24): a doubly labelled water study and repeated-measures analysis. J Nutr Sci. 10.1017/jns.2019.38 31501691 10.1017/jns.2019.20 PMC6722486

[103] Macdiarmid J, Blundell J (1998) Assessing dietary intake: who, what and why of under-reporting. Nutr Res Rev 11(2):231–253. 10.1079/NRR19980017 19094249 10.1079/NRR19980017

[104] Goris AH, Westerterp-Plantenga MS, Westerterp KR (2000) Undereating and underrecording of habitual food intake in obese men: selective underreporting of fat intake. Am J Clin Nutr 71:130–134. 10.1093/ajcn/71.1.130 10617957 10.1093/ajcn/71.1.130

[105] Livingstone MB, Prentice AM, Strain JJ et al (1990) Accuracy of weighed dietary records in studies of diet and health. Br Med J 300:708–712. 10.1136/bmj.300.6726.708 2386561 10.1136/bmj.300.6726.708 PMC1662510

