# Supplementary material for: Effects of energy-matched low- versus high-carbohydrate diets on glycaemic control, lipid profile, and body composition in healthy adults: a systematic review and meta-analysis of randomised controlled trials
Source: Eur J Nutr. 2026 Jan 6;65(1):19. doi: 10.1007/s00394-025-03862-z (PMC12775015; doi:10.1007/s00394-025-03862-z)
Supplement: Supplementary file 1 — Supplementary file1 (DOCX 42 KB) [file 394_2025_3862_MOESM1_ESM.docx]

# Supplementary Table S15. Full list of excluded studies with reasons for exclusion.

| **Study** | **Reason for exclusion** |
| --- | --- |
| Antonio J et al., 2016. The effects of a high protein diet on indices of health and body composition--a crossover trial in resistance-trained men | Not energy-matched groups |
| Appel LJ et al., 2005. Effects of protein, monounsaturated fat, and carbohydrate intake on blood pressure and serum lipids: results of the OmniHeart randomized trial | Both groups >45% CHO |
| Archer WR et al., 2003. Variations in body composition and plasma lipids in response to a high-carbohydrate diet | Both groups >45% CHO |
| Archer WR et al., 2005. Variations in plasma apolipoprotein C-III levels are strong correlates of the triglyceride response to a high-monounsaturated fatty acid diet and a high-carbohydrate diet | Both groups >45% CHO |
| Arciero PJ et al., 2008. Moderate protein intake improves total and regional body composition and insulin sensitivity in overweight adults | Both groups below 45% cho |
| Ashley JM et al., 2007. Nutrient adequacy during weight loss interventions: a randomized study in women comparing the dietary intake in a meal replacement group with a traditional food group | Both groups >45% CHO |
| Ashton EL et al., 2000. Diet high in monounsaturated fat does not have a different effect on arterial elasticity than a low-fat, high-carbohydrate diet | Not energy-matched |
| Ballesteros-Pomar MD et al., 2010. Effectiveness of energy-restricted diets with different protein:carbohydrate ratios: the relationship to insulin sensitivity | Not energy-matched |
| Baron JA et al., 1986. A randomized controlled trial of low carbohydrate and low fat/high fiber diets for weight loss | Not energy-matched |
| Bray GA et al., 2020. Effect of Overeating Dietary Protein at Different Levels on Circulating Lipids and Liver Lipid: The PROOF Study | Not energy-matched groups |
| Brinkworth GD et al., 2009. Effects of a low carbohydrate weight loss diet on exercise capacity and tolerance in obese subjects | Metabolic syndrome (it says that the participants had at least 2 metabolic syndrome risk factors) |
| Brinkworth GD et al., 2009. Long-term effects of a very-low-carbohydrate weight loss diet compared with an isocaloric low-fat diet after 12 mo | Metabolic syndrome |
| Brinkworth GD et al., 2016. Long-term effects of a very-low-carbohydrate weight-loss diet and an isocaloric low-fat diet on bone health in obese adults | Metabolic syndrome (it says that the participants had at least 2 metabolic syndrome risk factors) |
| Brown RC et al., 2000. High-carbohydrate versus high-fat diets: effect on body composition in trained cyclists | Not energy-matched |
| Brussaard JH et al., 1982. Serum lipoproteins of healthy persons fed a low-fat diet or a polyunsaturated fat diet for three months. A comparison of two cholesterol-lowering diets | Both groups >45% CHO |
| Bush et al., 2018. A high-fat compared with a high-carbohydrate breakfast enhances 24-hour fat oxidation in older adults. | Only breakfast differed in macronutrient composition; total daily diet not low-carbohydrate |
| Chien KY et al., 2024. High-protein diet with immediate post-exercise protein drink: Impact on appetite in middle-aged obesity | Not energy-matched groups |
| Claessens et al., 2009. The effect of a low-fat, high-protein or high-carbohydrate ad libitum diet on weight loss maintenance and metabolic risk factors. | Participants showed abnormally high insulin resistance at baseline (HOMA-IR > normal range) |
| Connor WE et al., 1991. Should a low-fat, high-carbohydrate diet be recommended for everyone? The case for a low-fat, high-carbohydrate diet | Review, no RCT |
| Crabtree et al., 2021. Comparison of ketogenic diets with and without ketone salts versus a low-fat diet: liver fat responses in overweight adults. | Not a randomized controlled trial for all groups (low-fat group non-randomized) |
| Dansinger ML et al., 2005. Comparison of the Atkins, Ornish, Weight Watchers, and Zone diets for weight loss and heart disease risk reduction: a randomized trial | Included current use of oral medication to treat hypertension, diabetes mellitus, or dyslipidemia. |
| De Luis et al., 2007. Effects of a low-fat versus a low-carbohydrate diet on adipocytokines in obese adults. | Both groups <45% CHO |
| De Luis et al., 2015. Effects of a high-protein/low-carbohydrate versus a standard hypocaloric diet on adipocytokine levels and insulin resistance in obese patients along 9 months. | Participants showed abnormally high insulin resistance at baseline (HOMA-IR > normal range) |
| Desroches S et al., 2006. Baseline plasma C-reactive protein concentrations influence lipid and lipoprotein responses to low-fat and high monounsaturated fatty acid diets in healthy men | Both groups >45% CHO |
| Due A et al., 2004. Effect of normal-fat diets, either medium or high in protein, on body weight in overweight subjects: a randomised 1-year trial | Both groups >45% CHO |
| Ebbeling CB et al., 2007. Effects of a low-glycemic load vs low-fat diet in obese young adults: a randomized trial | Not macro guidelines to participants |
| Ebbeling CB et al., 2018. Effects of a low carbohydrate diet on energy expenditure during weight loss maintenance: randomized trial | Same research group as the study of Ebbeling 2012; no relevant data |
| Ebbeling et al., 2022. Effects of a low-carbohydrate diet on insulin-resistant dyslipoproteinemia: a randomized controlled feeding trial. | Insufficient outcome data (no post-intervention SDs; only model-derived change estimates) |
| Fagerberg B et al., 1984. Weight-reducing diets: role of carbohydrates on sympathetic nervous activity and hypotensive response | No text |
| Farnsworth E et al., 2003. Effect of a high-protein, energy-restricted diet on body composition, glycemic control, and lipid concentrations in overweight and obese hyperinsulinemic men and women | Both groups >45% CHO |
| Foster GD et al., 1992. A controlled comparison of three very-low-calorie diets: effects on weight, body composition, and symptoms | Not energy-matched |
| Fleming J et al., 2003. Endurance capacity and high-intensity exercise performance responses to a high fat diet | Not energy-matched |
| Foster GD et al., 2003. A Randomized Trial of a Low-Carbohydrate Diet for Obesity | Not energy-matched |
| Foster GD et al., 2010. Weight and metabolic outcomes after 2 years on a low-carbohydrate versus low-fat diet: a randomized trial | Not energy-matched |
| Frisch et al., 2009. A randomized controlled trial on the efficacy of carbohydrate-reduced or fat-reduced diets in patients attending a telemedically guided weight loss program. | Participants had metabolic syndrome or metabolic risk factors at baseline |
| Gardner CD et al., 2007. Comparison of the Atkins, Zone, Ornish, and LEARN diets for change in weight and related risk factors among overweight premenopausal women: the A TO Z Weight Loss Study: a randomized trial | 32% Had metabolic syndrome |
| Gardner CD et al., 2018. Effect of Low-Fat vs Low-Carbohydrate Diet on 12-Month Weight Loss in Overweight Adults and the Association With Genotype Pattern or Insulin Secretion: The DIETFITS Randomized Clinical Trial | Taking medication was allowed |
| Gardner et al., 2016. Weight loss on low-fat vs low-carbohydrate diets by insulin resistance status among overweight adults and adults with obesity: a randomized pilot trial. | Participants met metabolic syndrome criteria at baseline |
| Golay A et al., 1996. Similar weight loss with low- or high-carbohydrate diets | Metabolic syndrome |
| Guevara-Cruz, M et al., 2024. Intermittent fasting, calorie restriction, and a ketogenic diet improve mitochondrial function by reducing lipopolysaccharide signaling in monocytes during obesity: A randomized clinical trial | Intervention < 4wks |
| Harvey-Berino J et al., 1999. Calorie restriction is more effective for obesity treatment than dietary fat restriction | Both groups >45% CHO |
| Henning PC et al., 2014. High protein diets do not attenuate decrements in testosterone and IGF-I during energy deficit | Intervention < 4wks |
| Hockaday TD et al., 1978. Prospective comparison of modified fat-high-carbohydrate with standard low-carbohydrate dietary advice in the treatment of diabetes: one year follow-up study | Diabetic patience |
| Hoover SE et al., 2021. Changes in Ghrelin and Glucagon following a Low Glycemic Load Diet in Women with PCOS | Not energy-matched groups |
| Hu et al., 2015. The effects of a low-carbohydrate diet vs a low-fat diet on novel cardiovascular risk factors: a randomized controlled trial. | Participants were taking lipid-lowering medication |
| Iglay HB et al., 2007. Resistance training and dietary protein: effects on glucose tolerance and contents of skeletal muscle insulin signaling proteins in older persons | Both groups >45% CHO |
| Johnston CS et al., 2004. High-protein, low-fat diets are effective for weight loss and favorably alter biomarkers in healthy adults | Insulin resistant QUICKI <0.339 at baseline |
| Johnston CS et al., 2006. Ketogenic low-carbohydrate diets have no metabolic advantage over nonketogenic low-carbohydrate diets | Both diets <45% CHO |
| Johnston CS et al., 2017. Use of Novel High-Protein Functional Food Products as Part of a Calorie-Restricted Diet to Reduce Insulin Resistance and Increase Lean Body Mass in Adults: A Randomized Controlled Trial | Permitted statins and hypertensive medication |
| Karl JP et al., 2015. Effects of carbohydrate quantity and glycemic index on resting metabolic rate and body composition during weight loss | Both groups >45% CHO |
| Keogh JB et al., 2008. Effects of weight loss from a very-low-carbohydrate diet on endothelial function and markers of cardiovascular disease risk in subjects with abdominal obesity | Hypertension lipid medication |
| Kleiner RE et al., 2006. Effects of an 8-week high-protein or high-carbohydrate diet in adults with hyperinsulinemia | Insulin resistant participants |
| Knopp RH et al., 1997. Long-term cholesterol-lowering effects of 4 fat-restricted diets in hypercholesterolemic and combined hyperlipidemic men. The Dietary Alternatives Study | Both groups >45% CHO |
| Labayen I et al., 2003. Effects of protein vs. carbohydrate-rich diets on fuel utilisation in obese women during weight loss. | No full-text |
| Lasker DA et al., 2008. Moderate carbohydrate, moderate protein weight loss diet reduces cardiovascular disease risk compared to high carbohydrate, low protein diet in obese adults: A randomized clinical trial | No post data |
| Layman DK et al., 2003. Increased dietary protein modifies glucose and insulin homeostasis in adult women during weight loss | No relevant data |
| Layman DK et al., 2009. A moderate-protein diet produces sustained weight loss and long-term changes in body composition and blood lipids in obese adults | Not energy-matched. >5% difference in calories between groups |
| Lean ME et al., 1997. Weight loss with high and low carbohydrate 1200 kcal diets in free living women | No macro breakdown or valid control of compliance |
| Leidy HJ et al., 2007. Higher protein intake preserves lean mass and satiety with weight loss in pre-obese and obese women | Both groups >45% CHO |
| Lim SS et al., 2010. Long-term effects of a low carbohydrate, low fat or high unsaturated fat diet compared to a no-intervention control | Taking meds included |
| Longland TM et al., 2016. Higher compared with lower dietary protein during an energy deficit combined with intense exercise promotes greater lean mass gain and fat mass loss: a randomized trial | Not energy-matched groups and Both groups >45% CHO |
| Luscombe-Marsh ND et al., 2005. Carbohydrate-restricted diets high in either monounsaturated fat or protein are equally effective at promoting fat loss and improving blood lipids | Both groups <45% CHO |
| Mamo JC et al., 2005. A low-protein diet exacerbates postprandial chylomicron concentration in moderately dyslipidaemic subjects in comparison to a lean red meat protein-enriched diet | Both groups >45% CHO |
| Martens EA et al., 2015. Maintenance of energy expenditure on high-protein vs. high-carbohydrate diets at a constant body weight may prevent a positive energy balance | No macro breakdown or valid control of compliance |
| McColley SP et al., 2011. A high-fat diet and the threonine-encoding allele (Thr54) polymorphism of fatty acid-binding protein 2 reduce plasma triglyceride-rich lipoproteins | Both groups >45% CHO |
| Meckling et al., 2007. A randomized trial of a hypocaloric high-protein diet, with and without exercise, on weight loss, fitness, and markers of the metabolic syndrome in overweight and obese women. | Dietary intake and compliance not adequately monitored across the 12-week intervention |
| Meckling KA et al., 2004. Comparison of a low-fat diet to a low-carbohydrate diet on weight loss, body composition, and risk factors for diabetes and cardiovascular disease in free-living, overweight men and women | Wrong population (5 had type 2 diabetes, 12 had abnormal BP, 6 had had hypercholesterolemia) |
| Michalczyk MM et al., 2020. The Effects of Low-Energy Moderate-Carbohydrate (MCD) and Mixed (MixD) Diets on Serum Lipid Profiles and Body Composition in Middle-Aged Men: A Randomized Controlled Parallel-Group Clinical Trial | Metabolic syndrome (high TAG 1.7mmol/l,low hdl <1.3mmol/l, high glucose >5.6mmol/l) |
| Mitchell CJ et al., 2017. The effects of dietary protein intake on appendicular lean mass and muscle function in elderly men: a 10-wk randomized controlled trial | Both groups >45% CHO |
| Mitra SR et al., 2019. Effect of an individualised high-protein, energy-restricted diet on anthropometric and cardio-metabolic parameters in overweight and obese Malaysian adults: a 6-month randomised controlled study | Both groups >45% CHO |
| Mojtahedi MC et al., 2011. The effects of a higher protein intake during energy restriction on changes in body composition and physical function in older women | Both groups >45% CHO |
| Noakes M et al., 2005. Effect of an energy-restricted, high-protein, low-fat diet relative to a conventional high-carbohydrate, low-fat diet on weight loss, body composition, nutritional status, and markers of cardiovascular health in obese women | Both groups >45% CHO |
| Katan MB et al., 1997. Should a low-fat, high-carbohydrate diet be recommended for everyone? Beyond low-fat diets | No text |
| Kogon MM et al., 1994. Psychological and metabolic effects of dietary carbohydrates and dexfenfluramine during a low-energy diet in obese women | No relevant data |
| OmniHeart Collaborative Research Group, 2005. Effects of protein, monounsaturated fat, and carbohydrate intake on blood pressure and serum lipids: results of the OmniHeart randomized trial. | All groups >45% CHO |
| Pasiakos SM et al., 2013. Effects of high-protein diets on fat-free mass and muscle protein synthesis following weight loss: a randomized controlled trial | Intervention < 4wks |
| Prins PJ et al., 2019. High Rates of Fat Oxidation Induced by a Low-Carbohydrate, High-Fat Diet, Do Not Impair 5-km Running Performance in Competitive Recreational Athletes | Not relevant outcomes |
| Rajaie et al., 2013. Comparative effects of carbohydrate versus fat restriction on serum levels of adipocytokines, markers of inflammation, and endothelial function among women with the metabolic syndrome: a randomized cross-over clinical trial. | Participants had metabolic syndrome |
| Rasmussen et al., 2007. Effect on 24-h energy expenditure of a moderate-fat diet high in monounsaturated fatty acids compared with that of a low-fat, carbohydrate-rich diet: a 6-mo controlled dietary intervention trial. | Energy intake not matched between groups (>200 kcal difference) |
| Rietman A et al., 2014. Increasing protein intake modulates lipid metabolism in healthy young men and women consuming a high fat hypercaloric diet | Intervention < 4 |
| Sacks et al., 2014. Effects of high vs low glycemic index of dietary carbohydrate on cardiovascular disease risk factors and insulin sensitivity: the OmniCarb randomized clinical trial. | Participants met metabolic syndrome criteria at baseline |
| Sacks FM et al., 2009. Comparison of weight-loss diets with different compositions of fat, protein, and carbohydrates | Both groups >45% CHO |
| Samaha et al., 2003. A low-carbohydrate as compared with a low-fat diet in severe obesity. | Participants had diabetes and/or metabolic syndrome at baseline |
| Schutte S et al., 2022. Diverging metabolic effects of 2 energy-restricted diets differing in nutrient quality: a 12-week randomized controlled trial in subjects with abdominal obesity | Both groups >45% CHO |
| Scott CB et al., 1992. Effect of macronutrient composition of an energy-restrictive diet on maximal physical performance | Six women were using oral contraseptives |
| Sharman MJ et al., 2002. A Ketogenic Diet Favorably Affects Serum Biomarkers for Cardiovascular Disease in Normal-Weight Men | Not RCT (groups allocated based on preference of participants) |
| Sharman MJ et al., 2004. Very low-carbohydrate and low-fat diets affect fasting lipids and postprandial lipemia differently in overweight men | Not energy-matched |
| Skov AR et al., 1999. Randomized trial on protein vs carbohydrate in ad libitum fat reduced diet for the treatment of obesity | Both groups >45% CHO |
| Soenen S et al., 2012. Relatively high-protein or 'low-carb' energy-restricted diets for body weight loss and body weight maintenance? | Not energy-matched groups |
| Stern L et al., 2004. The effects of low-carbohydrate versus conventional weight loss diets in severely obese adults: one-year follow-up of a randomized trial | Not energy-matched |
| Sun J et al., 2023. The effect of dietary carbohydrate and calorie restriction on weight and metabolic health in overweight/obese individuals: a multi-center randomized controlled trial | INSULIN resistant participants homa >2.9 |
| Tang M et al., 2013. Normal vs. high-protein weight loss diets in men: effects on body composition and indices of metabolic syndrome | Both groups >45% CHO |
| Tang M et al., 2013. Regional, but not total, body composition changes in overweight and obese adults consuming a higher protein, energy-restricted diet are sex specific | Both groups >45% CHO |
| Teng et al., 2017. Effects of exchanging carbohydrate or monounsaturated fat with saturated fat on inflammatory and thrombogenic responses in subjects with abdominal obesity: a randomized controlled trial. | Both groups >45% CHO |
| Thomson et al., 2010. Changes in body weight and metabolic indexes in overweight breast cancer survivors enrolled in a randomized trial of low-fat vs. reduced carbohydrate diets. | Participants were breast cancer survivors with prior treatment (medical condition) |
| Truby H et al., 2006. Randomised controlled trial of four commercial weight loss programmes in the UK: initial findings from the BBC "diet trials" | Not energy-matched |
| Turley ML et al., 1998. The effect of a low-fat, high-carbohydrate diet on serum high density lipoprotein cholesterol and triglyceride | Not energy-matched groups |
| Vargas-Molina S et al., 2021. Effects of a low-carbohydrate ketogenic diet on health parameters in resistance-trained women | Not energy-matched groups |
| Veum et al., 2017. Visceral adiposity and metabolic syndrome after very high-fat and low-fat isocaloric diets: a randomized controlled trial. | Participants metabolically impaired / insulin-resistant at baseline |
| Volek JS et al., 2002. Body composition and hormonal responses to a carbohydrate-restricted diet | Not energy-matched groups (205kcal difference) |
| Volek JS et al., 2003. An isoenergetic very low carbohydrate diet improves serum HDL cholesterol and triacylglycerol concentrations, the total cholesterol to HDL cholesterol ratio and postprandial pipemic responses compared with a low fat diet in normal weight, normolipidemic women | Not energy-matched |
| Volek JF et al., 2004. Comparison of a very low-carbohydrate and low-fat diet on fasting lipids, LDL subclasses, insulin resistance, and postprandial lipemic responses in overweight women | Intervention <4wks |
| Volek JS et al., 2009. Carbohydrate restriction has a more favorable impact on the metabolic syndrome than a low fat diet | Atherogenic Dyslipidemia |
| Volek JS et al., 2000. Fasting Lipoprotein and Postprandial Triacylglycerol Responses to a Low-Carbohydrate Diet Supplemented with n-3 Fatty Acids | Not energy-matched |
| Wal JS et al., 2007. Moderate-carbohydrate low-fat versus low-carbohydrate high-fat meal replacements for weight loss | Not energy-matched |
| Weigle DS et al., 2005. A high-protein diet induces sustained reductions in appetite, ad libitum caloric intake, and body weight despite compensatory changes in diurnal plasma leptin and ghrelin concentrations | Both groups >45% CHO |
| Wycherley TP et al., 2013. Comparison of the effects of weight loss from a high-protein versus standard-protein energy-restricted diet on strength and aerobic capacity in overweight and obese men | 64% had metabolic syndrome |
| Yancy WS Jr et al., 2004. A low-carbohydrate, ketogenic diet versus a low-fat diet to treat obesity and hyperlipidemia: a randomized, controlled trial | Insufficient data (outcomes reported only as model-derived estimates, no raw SDs) |
| Young LR et al., 2011. Effect of dietary fat and omega-3 fatty acids on urinary eicosanoids and sex hormone concentrations in postmenopausal women: a randomized controlled feeding trial | Both groups >45% CHO |
| Zajac A et al., 2014. The effects of a ketogenic diet on exercise metabolism and physical performance in off-road cyclists | Only acute effects during exercise measured |
