# Supplementary material for: Effects of energy-matched low- versus high-carbohydrate diets on glycaemic control, lipid profile, and body composition in healthy adults: a systematic review and meta-analysis of randomised controlled trials
Source: Eur J Nutr. 2026 Jan 6;65(1):19. doi: 10.1007/s00394-025-03862-z (PMC12775015; doi:10.1007/s00394-025-03862-z)
Supplement: Supplementary file 2 — Supplementary file2 (DOCX 30 KB) [file 394_2025_3862_MOESM2_ESM.docx]

**Table S1. Risk of Bias assessment of included randomized controlled trials (parallel and crossover designs)**

Risk of bias was assessed using the Cochrane RoB 2.0 tool across five domains (D1–D5) and summarized as an overall judgement. Symbols and colors indicate the domain-level judgement: '+' = Low risk (green), '–' = Some concerns (yellow), 'X' = High risk (red). For crossover trials, carryover was assessed as part of the design-specific RoB tool and reflected in the Overall judgement.

| **Study** | **Design** | **D1 Randomization** | **D2 Deviations** | **D3 Missing data** | **D4 Outcome measurement** | **D5 Selective reporting** | **Overall** |
| --- | --- | --- | --- | --- | --- | --- | --- |
| Morgan 2008 | Parallel | + | + | + | + | + | + |
| Bazzano 2014 | Parallel | + | + | + | + | + | + |
| Das 2007 | Parallel | + | + | + | + | + | + |
| Keogh 2007 | Parallel | + | + | + | + | + | + |
| Brehm 2003 | Parallel | + | + | + | + | + | + |
| Volek 2004 | Crossover | + | + | + | + | + | + |
| Cornier 2005 | Parallel | + | + | + | + | + | + |
| Buga 2022 | Crossover | + | + | + | + | + | + |
| Wilson 2017 | Parallel | + | + | + | + | + | + |
| Layman 2005 | Parallel | + | + | + | + | + | + |
| Parr 2016 | Parallel | + | + | + | + | + | + |
| Racette 1995 | Parallel | + | + | + | + | + | + |
| Green 2018 | Crossover | + | + | + | + | + | + |
| Walberg 1988 | Parallel | + | + | + | + | + | + |
| Paoli 2021 | Parallel | + | + | + | + | + | + |
| Layman 2003 | Parallel | + | + | + | + | + | + |
| Ebbeling 2012 | Crossover | + | + | + | + | + | + |
| Summer 2011 | Parallel | + | + | + | + | + | + |

**Legend**

| **Symbol** | **Meaning** |
| --- | --- |
| + | Low risk |
| – | Some concerns |
| X | High risk |

Notes: D1 = Randomization process; D2 = Deviations from intended interventions; D3 = Missing outcome data; D4 = Measurement of the outcome; D5 = Selection of the reported result. Carryover was assessed for crossover trials (Buga 2022; 2018; Ebbeling 2012; Volek 2004) using the RoB 2.0 crossover extension and is reflected in the Overall judgement.
