# Supplementary material for: Effects of energy-matched low- versus high-carbohydrate diets on glycaemic control, lipid profile, and body composition in healthy adults: a systematic review and meta-analysis of randomised controlled trials
Source: Eur J Nutr. 2026 Jan 6;65(1):19. doi: 10.1007/s00394-025-03862-z (PMC12775015; doi:10.1007/s00394-025-03862-z)
Supplement: Supplementary file 3 — Supplementary file3 (DOCX 29 KB) [file 394_2025_3862_MOESM3_ESM.docx]

| **Outcome** | **Effect (Direction & Magnitude)** | **No. of Studies (Participants)** | **Risk of Bias** | **Inconsistency** | **Indirectness** | **Imprecision** | **Publication Bias** | **Overall Certainty** | **Summary of Findings** |
| --- | --- | --- | --- | --- | --- | --- | --- | --- | --- |
| Fasting Blood Glucose (FBG) | ↓ LC vs HC (g = –0.33; P = 0.001) | 11 (486) | Low | Low | None | Low | Low | High | LC diets modestly lower fasting glucose under isocaloric conditions. |
| Fasting Insulin (FINS) | ↓ LC vs HC (g = –0.19; P = 0.039) | 11 (484) | Low | Moderate | None | Moderate | Low | Moderate–High | Small but significant reduction in insulin levels under LC diets. |
| Total Cholesterol (TC) | No difference (g = –0.15; P = 0.10) | 13 (428) | Low | Low | None | Moderate | Low | Low | No clear effect of diet type on TC. |
| Triglycerides (TAG) | ↓ LC vs HC (g = –0.43; P < 0.001) | 15 (585) | Low | Low | None | Low | Possible bias | High | Consistent and clinically relevant TAG reduction with LC diets. |
| LDL-C | ↓ HC vs LC (g = –0.23; P = 0.004) | 14 (560) | Low | Low | None | Low | Low | High | HC diets produce slightly greater LDL-C reduction. |
| HDL-C | ↑ LC vs HC (g = 0.41; P < 0.001) | 15 (585) | Low | Low | None | Low | Possible bias | High | LC diets consistently raise HDL-C. |
| Body Mass (BM) | ↓ LC vs HC (g = –0.18; P = 0.023) | 12 (542) | Low | Low | None | Moderate | Low | High | Slight but significant BM decrease with LC diets. |
| Fat Mass (FM) | ↓ LC vs HC (g = –0.28; P = 0.011) | 7 (271) | Low | Low–Moderate | None | Moderate | Possible bias | Moderate–High | LC diets reduce FM without affecting lean mass. |
| Fat-Free Mass (FFM) | No difference (g = 0.11; P = 0.39) | 5 (171) | Low | Low | None | High | Low | Low | Neither diet affects FFM under isocaloric conditions. |

**Supplementary Table S14. GRADE Summary of Evidence Certainty for Main Outcomes**

Notes: Certainty ratings follow the GRADE approach (high, moderate, low, very low) and reflect assessment of methodological quality, consistency, precision, directness, and publication bias for each outcome. Ratings are based on pooled meta-analytic results reported in the main manuscript.
