# Supplementary figures and images for: Effects of energy-matched low- versus high-carbohydrate diets on glycaemic control, lipid profile, and body composition in healthy adults: a systematic review and meta-analysis of randomised controlled trials
Source: Eur J Nutr. 2026 Jan 6;65(1):19. doi: 10.1007/s00394-025-03862-z (PMC12775015; doi:10.1007/s00394-025-03862-z)

# Fasting Blood Glucose

**Funnel Plot of Standard Error by Hedges's g**

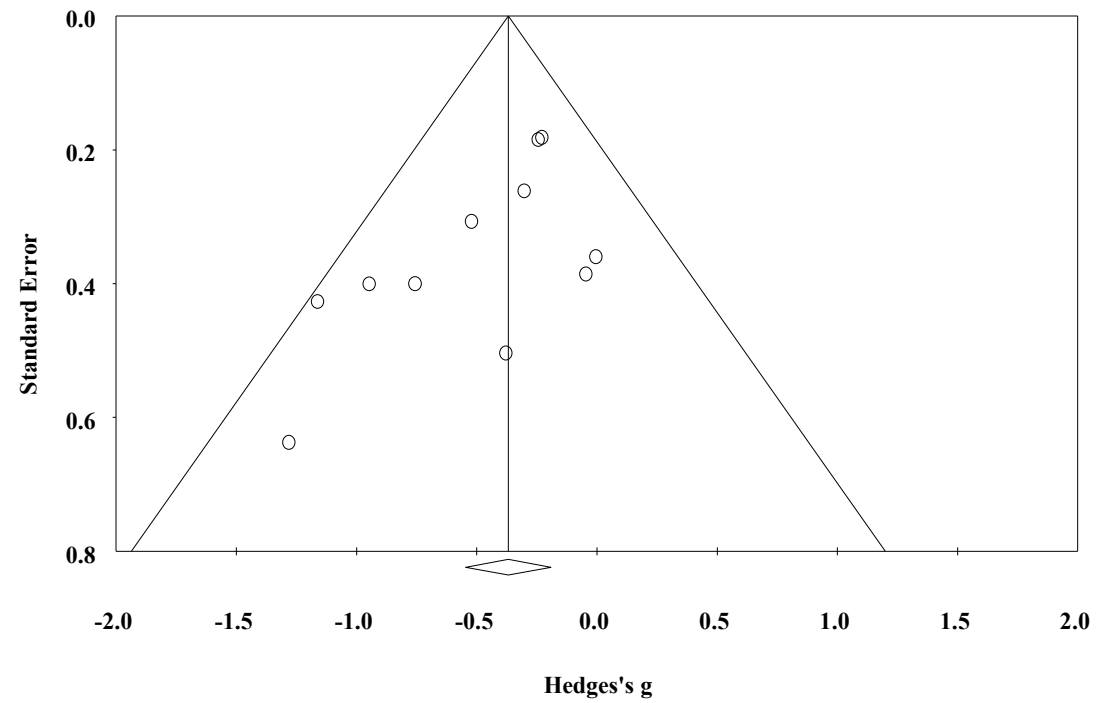

Supplement: Supplementary file 4 — Supplementary file4 (PDF 56 KB) [file 394_2025_3862_MOESM4_ESM.pdf]

# Fasting Insulin

**Funnel Plot of Standard Error by Hedges's g**

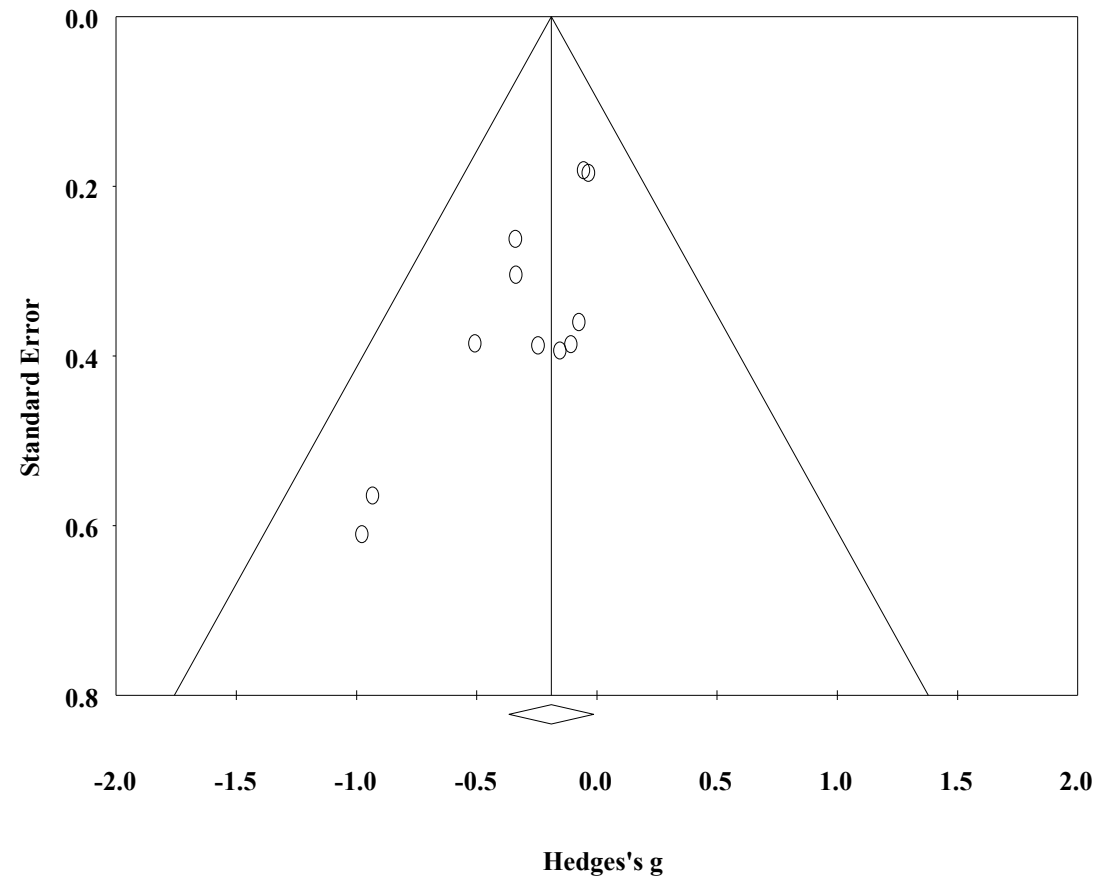

Supplement: Supplementary file 5 — Supplementary file5 (PDF 55 KB) [file 394_2025_3862_MOESM5_ESM.pdf]

# Total Cholesterol

**Funnel Plot of Standard Error by Hedges's g**

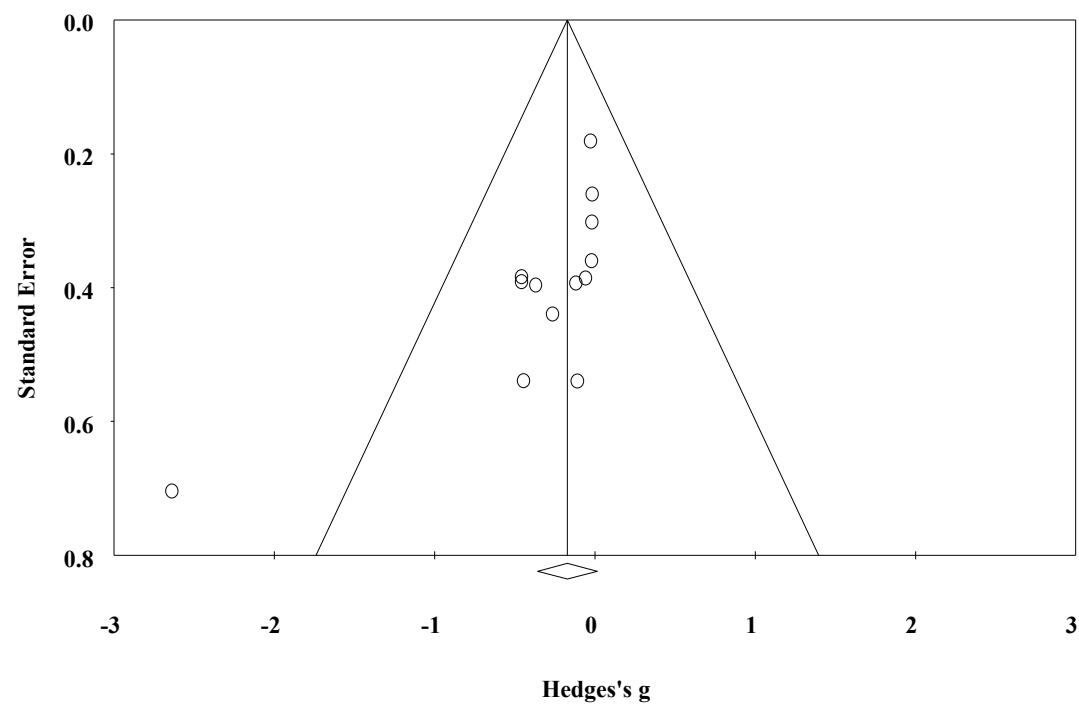

Supplement: Supplementary file 6 — Supplementary file6 (PDF 55 KB) [file 394_2025_3862_MOESM6_ESM.pdf]

# Triglycerides

**Funnel Plot of Standard Error by Hedges's g**

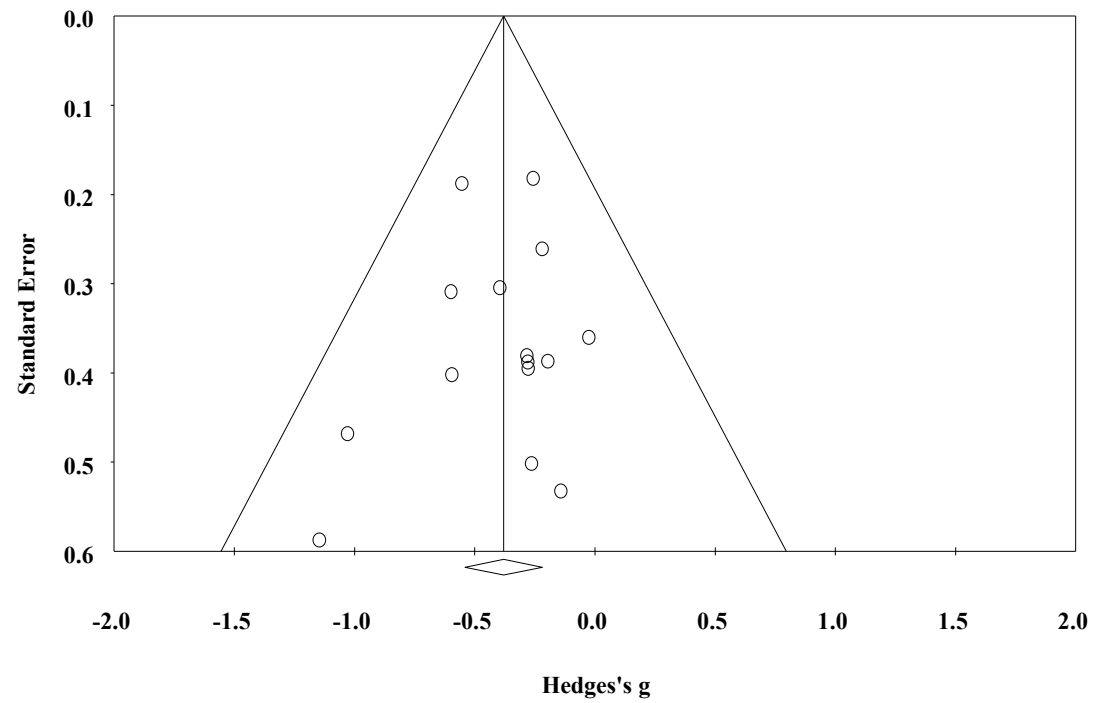

Supplement: Supplementary file 7 — Supplementary file7 (PDF 56 KB) [file 394_2025_3862_MOESM7_ESM.pdf]

LDL-Cholesterol

**Funnel Plot of Standard Error by Hedges's g**

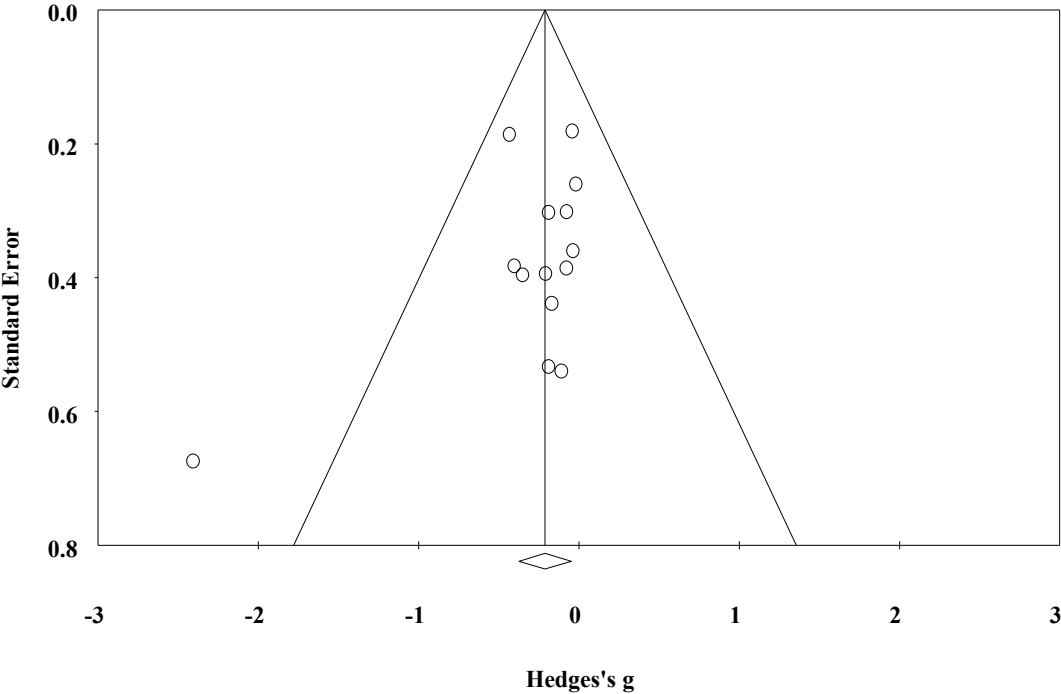

Supplement: Supplementary file 8 — Supplementary file8 (PDF 55 KB) [file 394_2025_3862_MOESM8_ESM.pdf]

# HDL-Cholesterol

**Funnel Plot of Standard Error by Hedges's g**

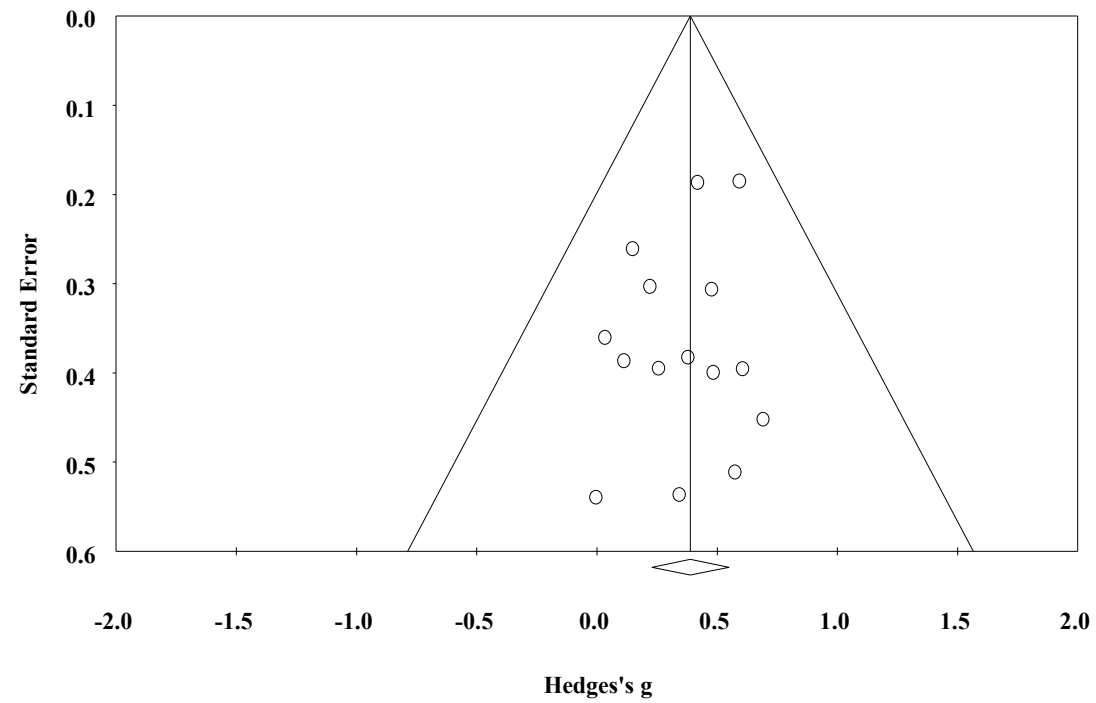

Supplement: Supplementary file 9 — Supplementary file9 (PDF 56 KB) [file 394_2025_3862_MOESM9_ESM.pdf]

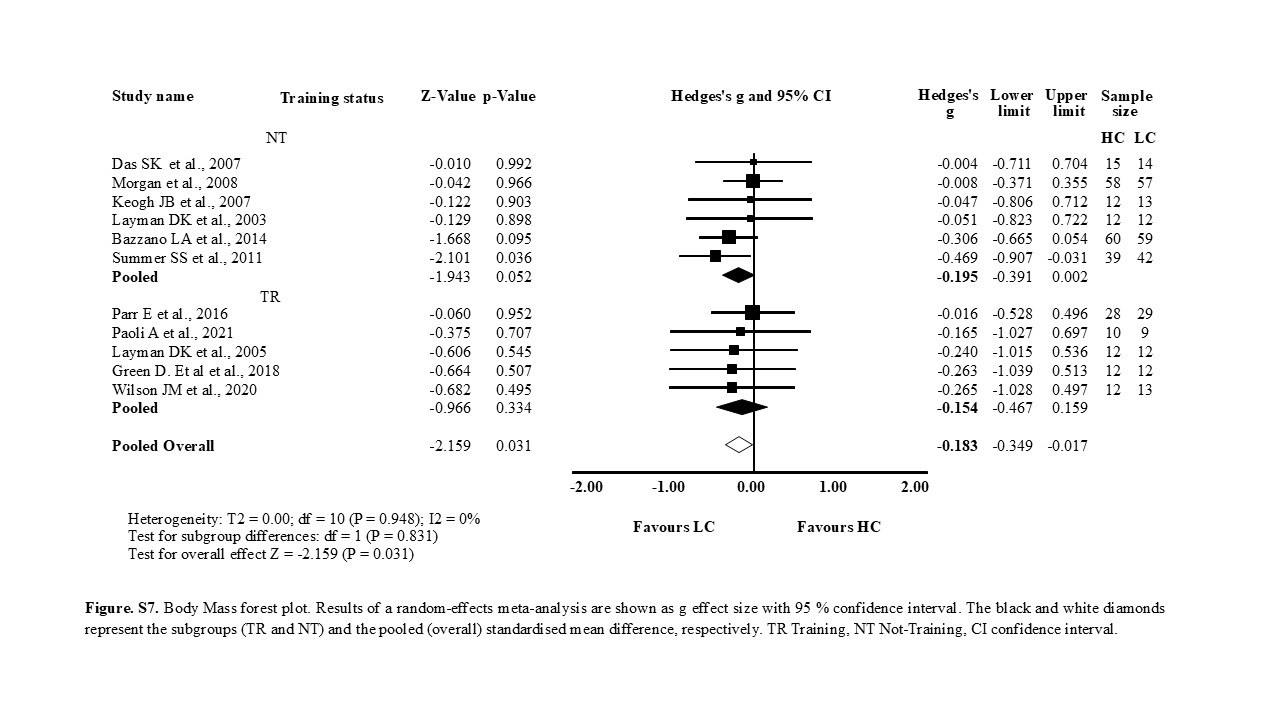

Supplement: Supplementary file 10 — Supplementary file10 (JPG 140 KB) [file 394_2025_3862_MOESM10_ESM.jpg]

# Body Mass

**Funnel Plot of Standard Error by Hedges's g**

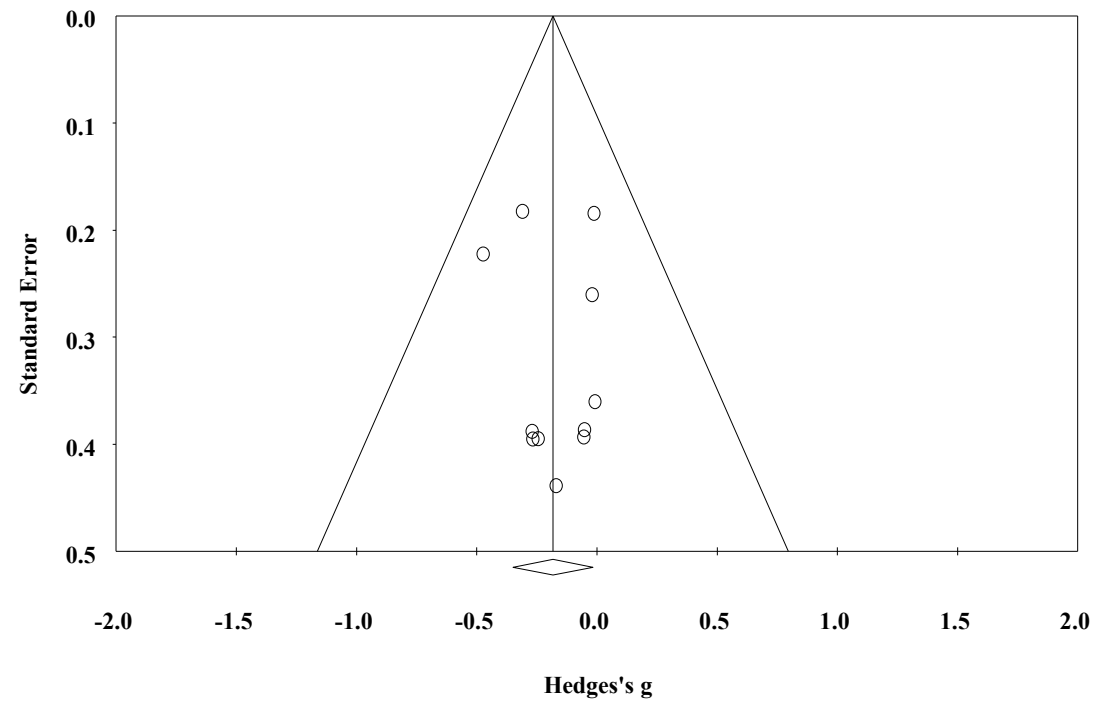

Supplement: Supplementary file 11 — Supplementary file11 (PDF 54 KB) [file 394_2025_3862_MOESM11_ESM.pdf]

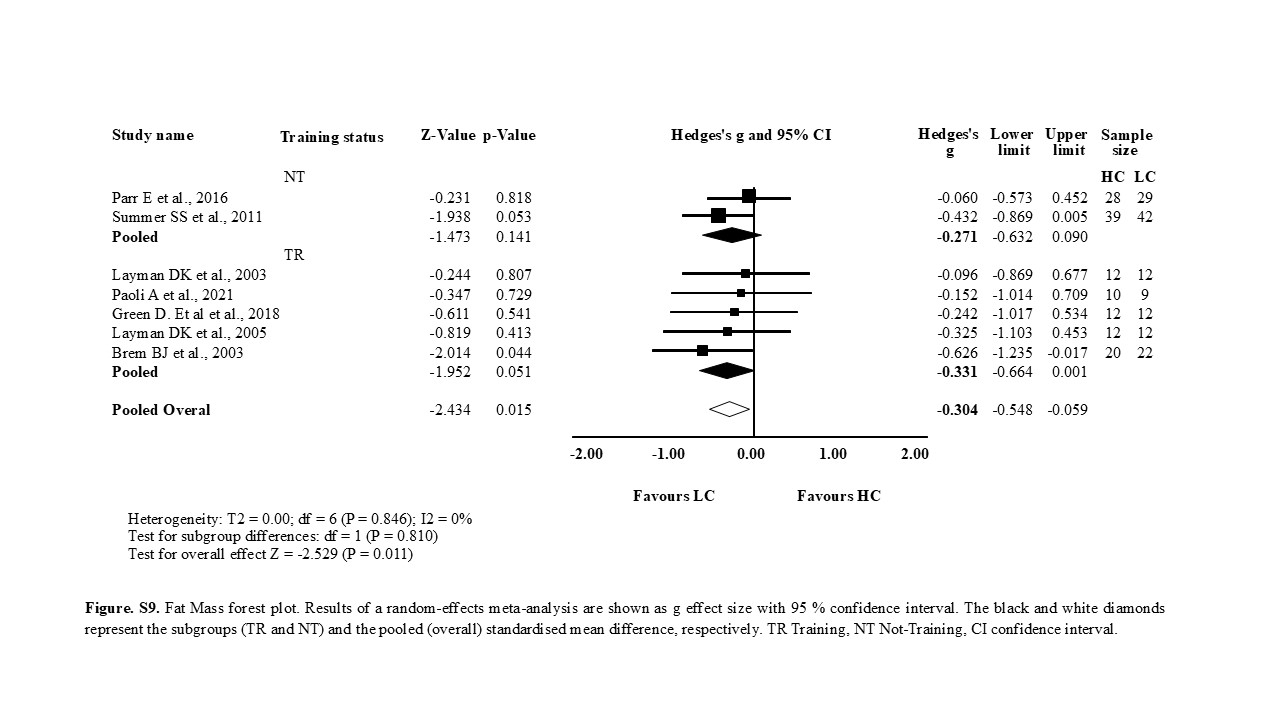

Supplement: Supplementary file 12 — Supplementary file12 (JPG 118 KB) [file 394_2025_3862_MOESM12_ESM.jpg]

## Fat Mass

**Funnel Plot of Standard Error by Hedges's g**

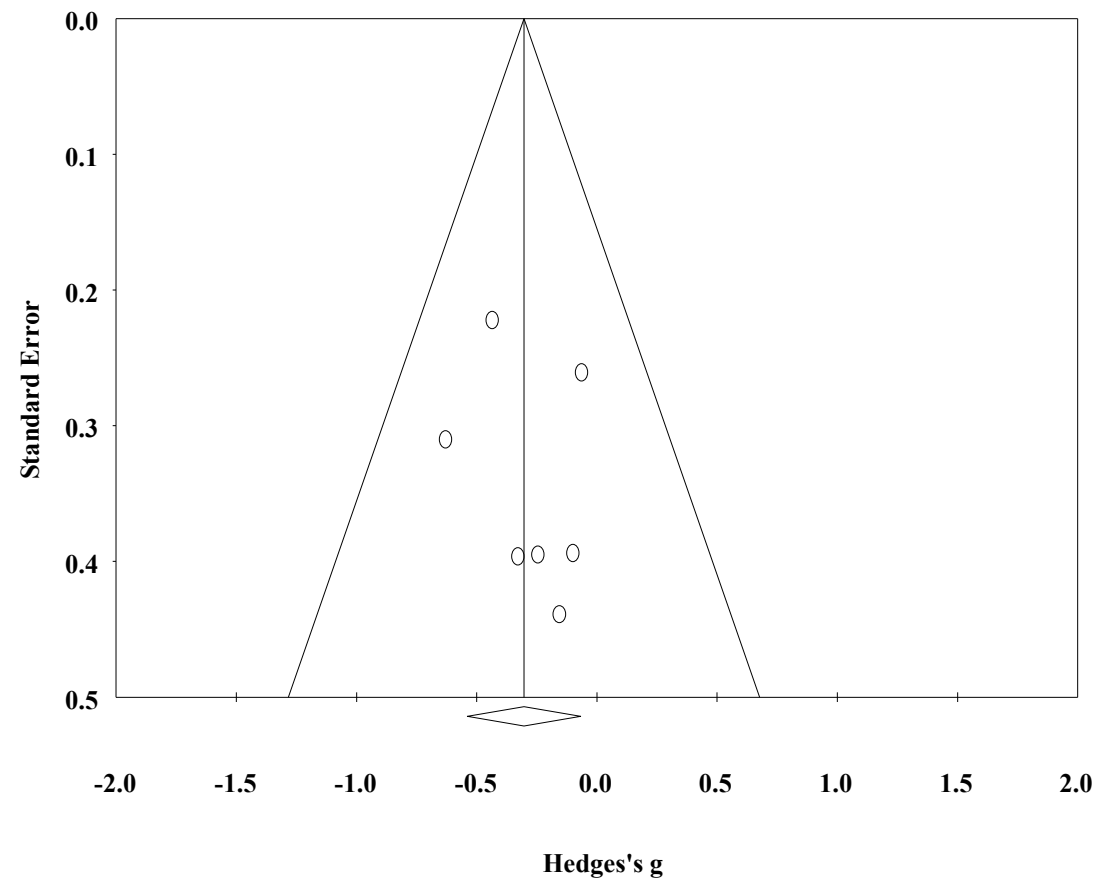

Supplement: Supplementary file 13 — Supplementary file13 (PDF 53 KB) [file 394_2025_3862_MOESM13_ESM.pdf]

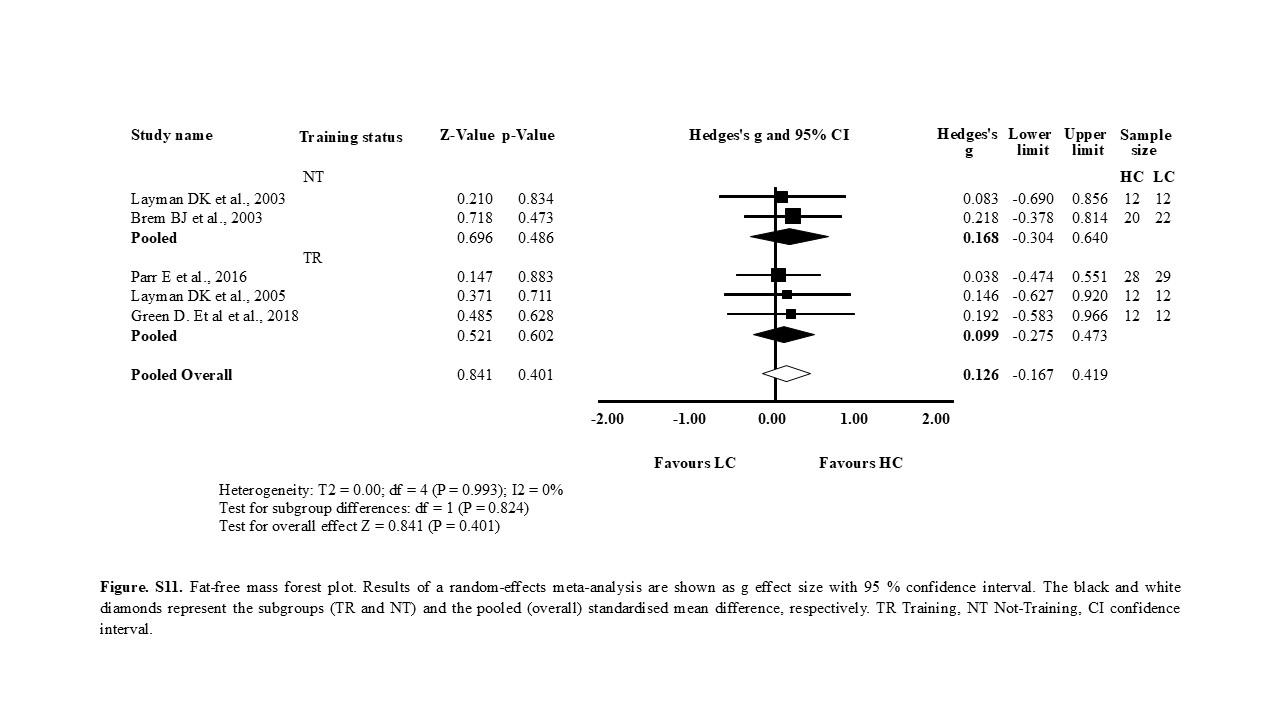

Supplement: Supplementary file 14 — Supplementary file14 (JPG 105 KB) [file 394_2025_3862_MOESM14_ESM.jpg]

# Fat-free Mass

**Funnel Plot of Standard Error by Hedges's g**

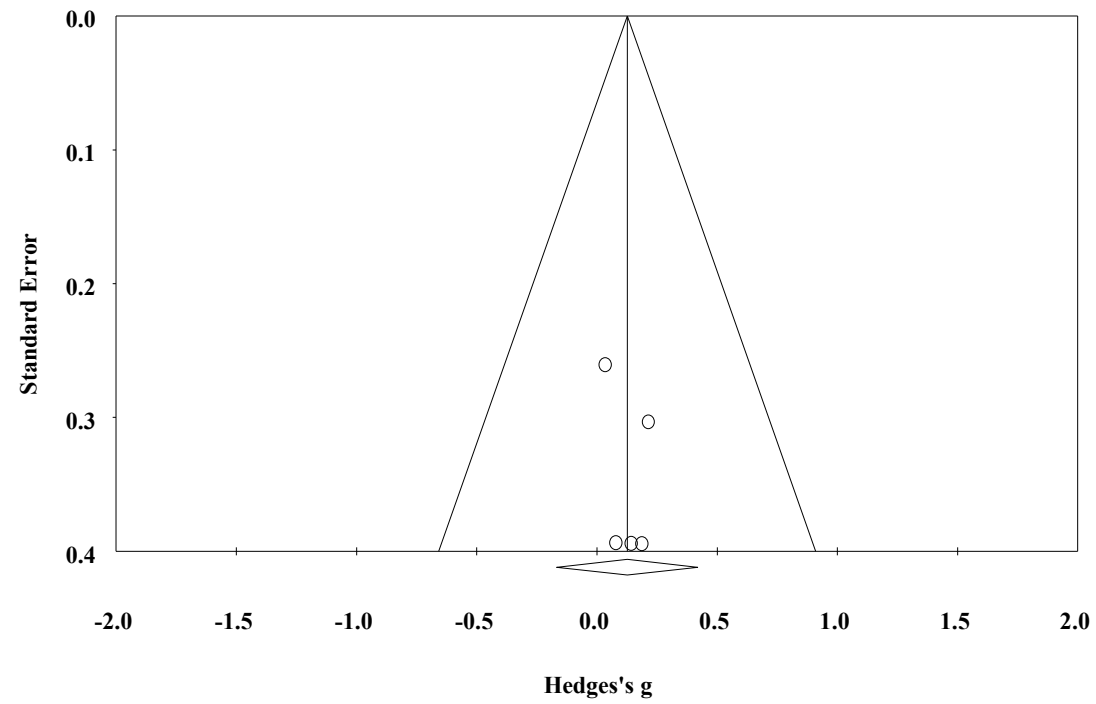

Supplement: Supplementary file 15 — Supplementary file15 (PDF 52 KB) [file 394_2025_3862_MOESM15_ESM.pdf]
